# Syndecan-4 in microglia mediates ischemic stroke-induced mitochondrial dysfunction and blood-brain barrier damage by interacting with Dishevelled

**DOI:** 10.1038/s41598-026-50594-z

**Published:** 2026-05-29

**Authors:** Dongya Chen, Dan Luo, Yuxing Wei

**Affiliations:** 1 School of Basic Medicine and Clinical Pharmacy and State Key Laboratory of Natural Medicines, China Pharmaceutical University, Nanjing, China; 2 National Key Laboratory of Immunity and Inflammation, Suzhou Institute of Systems Medicine, Chinese Academy of Medical Sciences, Suzhou, China

**Keywords:** Ischemia/reperfusion, Syndecan-4, Dishevelled, Mitochondria, Blood-brain barrier, Cell biology, Diseases, Neurology, Neuroscience

## Abstract

Ischemic stroke is a leading cause of disability and mortality, resulting in impaired mitochondrial function and disruption of the blood-brain barrier (BBB). Studies have demonstrated that Syndecan-4 (SDC4) influences BBB integrity and function, however, it remains unclear whether SDC4 influences mitochondrial function and BBB integrity following a stroke. We first obtained single-cell data from cortical tissue of transient middle cerebral artery occlusion (tMCAO) mice from public databases to identify changes in disease-associated cells and related molecular composition during disease progression. We then performed functional analyses to elucidate the functional characteristics of microglial subsets. Trajectory analysis was used to investigate cellular transition state signatures. Subsequently, we injected adeno-associated virus-sh_SDC4 (AAV-sh_SDC4) into the brain of C57BL/6J mice before tMCAO. Primary microglia were cultured and transfected with lentiviral-sh_SDC4 (LV-sh_SDC4) before oxygen-glucose deprivation/reoxygenation (OGD/R). Western blot, flow cytometry, and quantitative reverse transcription-polymerase chain reaction (qRT-PCR) were used to investigate the expression, function, and mechanisms of SDC4. Molecular docking and molecular dynamics simulations were used to investigate binding. We identified 13 major cell populations, most of which underwent dynamic changes after MCAO. Microglia were the predominant cell population in all groups. Subsequent clustering analysis demonstrated that the relative abundances of repair- and anti-inflammatory as well as senescence-related microglial subpopulations were reduced following MCAO. Complementary trajectory inference modeling further uncovered a progressive upregulation of SDC4 expression in microglia throughout the course of disease progression. To validate these observations, we assessed SDC4 levels in experimental models of cerebral ischemia-reperfusion (I/R) injury and found elevated expression of this protein in both in vitro and in vivo settings. AAV-sh_SDC4 significantly improved I/R-induced motor impairment, restored mitochondrial morphology and function, increased occludin and claudin-5 expression, and protected BBB integrity. AAV-sh_SDC4 and LV-sh_SDC4 treatment enhanced Wnt/β-catenin signaling. XAV-939 reversed the protective effects of SDC4 interference. Mechanistically, SDC4 interacts with Dishevelled (Dvl) in microglia, physically sequestering Dvl and inhibiting Wnt/β-catenin signaling, ultimately leading to microglial mitochondrial dysfunction and associated BBB damage. The interaction between SDC4 and Dvl promotes mitochondrial dysfunction in microglia and is closely associated with the disruption of BBB integrity, thus offering a potential therapeutic strategy for the clinical treatment of cerebral ischemia.

## Introduction

The high mortality and disability rates associated with ischemic stroke are closely related to the neurological deficits induced by cerebral ischemia. Numerous studies have demonstrated that ischemic stroke leads to neuronal damage, elevated levels of inflammatory factors, mitochondrial structure and function damage and impaired blood-brain barrier (BBB)^1^. The area around the cerebral ischemic nucleus is not a significant nerve death and can be preserved after blood is restored^2,3^. Therefore, identifying an effective target to counteract the neuronal damage induced by ischemia-reperfusion (I/R) is of significant importance.

Microglia is the most important brain immune cell and is the main protection of brain activity. They provide several metabolic conditions for exogenous stimulation^4,5^. Microglia is distributed throughout the brain, which makes the brain count relatively constant in adults^6,7^. Microglia continuously survey the brain microenvironment to maintain the brain through the expansion and depletion process^8^. In states of acute exhaustion or illness, microglia rapidly proliferate and clonally expand their numbers, thereby restoring the homeostatic pool^9^ or forming microglial clusters around the lesion site^10^. Upon cerebral ischemia, microglia undergo activation, this activation is characterized by the production of reactive oxygen species (ROS) and pro-inflammatory cytokines, which can exacerbate damage to endothelial cells, leading to increased permeability of the BBB^11,12^.Excessive ROS affects microglial activation states, and contributes to BBB disruption by modulating matrix metalloproteinases (MMPs), further degrading tight junction proteins critical for maintaining the barrier^13^. This breach allows for increased permeability, facilitating neuronal damage through excitotoxicity and edema due to a lack of homeostasis in ion and water balance^14^. Activated microglia can adopt either a pro-inflammatory M1 phenotype, which exacerbates BBB disruption, or an anti-inflammatory M2 phenotype, which supports repair mechanisms and promotes neuron survival^15^. The balance between these instances of detrimental and beneficial effects is influenced by the microglial phenotype. Currently, there is limited understanding of the specific molecular determinants that regulate the structure and function of microglial mitochondria and the integrity of the blood-brain barrier following cerebral infarction.

The syndecan family-comprising four distinct isoforms (SDC1 through SDC4)-is a group of cell membrane-associated heparan sulfate proteoglycans that are widely distributed across various tissues in mammalian organisms^16^. Syndecan-4 (SDC4) is a transmembrane proteoglycan critically involved in multiple signaling cascades, such as those governing tumor progression, angiogenesis, and wound healing^17^. Notably, SDC4 serves as a marker of inflammatory activity in senescent endothelial cells. Based on these roles, we hypothesize that SDC4 represents a promising therapeutic target for ischemic stroke. Previous studies have reported that hypoxia induces SDC4 upregulation across diverse cell types-including human retinal pigment epithelial cells, dermal fibroblasts, myeloid cells, and colon carcinoma cells^18,19^-suggesting its potential function as an oxygen-sensitive protein. Literature has shown that SDC4 knockout mice exhibit angiogenesis disorders^20^. The expression of syndecan-4 (SDC4) is additionally critical for the functional integrity of inflammatory cells that populate the tumor microenvironment. A prior investigation by Wang et al. demonstrated that SDC4 acts as a coreceptor for tumor necrosis factor-α (TNF-α). In addition, transforming growth factor-β (TGF-β) inhibits TNF-α-driven MMP-3 synthesis by downregulating both SDC4 activity and nuclear factor-κB (NF-κB) signaling cascades. Importantly, within macrophage subsets, TNF-α directly increases the expression levels of SDC4^21,22^. Furthermore, existing literature has well-documented upregulated expression of two syndecan isoforms-neuron-specific SDC3 and broadly expressed SDC4-in post-mortem human Alzheimer’s disease (AD) brain tissues and APP/PS1 transgenic mouse models of AD^23^. In alignment with this, our analysis of transcriptomic sequencing data from a murine model of ischemic stroke revealed elevated SDC4 expression in microglia following middle cerebral artery occlusion. These findings suggest that SDC4 may be involved in regulating microglial dysfunction and BBB integrity under ischemic conditions.

In this study, we conducted a comprehensive analysis of scRNA-seq data from previous investigations, with the aim of capturing and examining the cellular and molecular adaptive changes associated with MCAO at a single-cell resolution. We investigated the pathological role of SDC4 in mitochondrial dysfunction and blood-brain barrier disruption following microglia-induced MCAO. Our results indicate that SDC4 expression is upregulated in microglia after ischemia-reperfusion in mice. The interaction between SDC4 and Dishevelled (Dvl) facilitates the sequestration of Dvl, which in turn inhibits the activation of the Wnt/β-catenin pathway, compromising mitochondrial structure and function, which is closely associated with the disruption of blood-brain barrier integrity. Reduced SDC4 expression enhances neurological function in MCAO mice restores mitochondrial structure and function, and protects the blood-brain barrier. Our findings suggest that SDC4 plays a crucial role in shaping microglial function and maintaining brain homeostasis.

## Materials and methods

### Data acquisition

The Gene Expression Omnibus (GEO)-a public repository for gene expression data-has been developed and curated by the U.S. National Center for Biotechnology Information (NCBI). Specifically, the single-cell transcriptomic dataset GSE227651 was retrieved from this NCBI-hosted GEO repository. This file contains four samples, including one sham group and three tMCAO samples. The tMCAO samples were divided into 1, 3, and 7-days groups.

### Quality control of single-cell data

At the outset, gene expression profiles were imported via the Seurat computational package, where we applied cell filtering criteria including per-cell total UMI counts, number of expressed genes per cell, and per-cell mitochondrial gene expression percentage, thresholds were defined as nFeature_RNA > 200 and percent.mt < 5. Next, we utilized DoubletFinder software (version 2.0.4) to eliminate doublet cells for each sample separately, thus finalizing the cellular quality control workflow.

### Animals

Adult male C57BL/6J mice (aged 6–7 weeks and weighing 22–26 g) were sourced from China Pharmaceutical University. The animals were housed under a 12/12-hour light/dark cycle at a constant temperature of 25 °C. The Animal Ethics Committee of the China Pharmaceutical University granted approval for all experimental procedures (Approval No. YSL-202506116). All animals used in this study were fed and managed in accordance with the Laboratory Animal Husbandry and Management Standards of China Pharmaceutical University and the ARRIVE guidelines.

### Surgical procedure

To induce focal ischemic stroke in adult male C57BL/6J mice, we employed the transient middle cerebral artery occlusion (tMCAO) model as previously described^24^. Adult male C57BL/6J mice were administered pentobarbital sodium anesthesia (dosed at 35 mg per kg of body weight) via intraperitoneal (i.p.) injection into the lower right ventral abdominal quadrant. An incision was made along the midline of the neck to access and ligate the right common and external carotid arteries. A microvascular clip was applied to the internal carotid artery, and a silicone-tipped occlusion filament with a diameter of 0.20 mm from RWD Life Science (Shenzhen, China) was inserted into the artery until slight resistance was encountered (approximately 9–11 mm). Following a 90-minute occlusion duration, the filament was removed to restore cerebral blood flow (reperfusion) under aseptic surgical conditions. For the Sham control group, all anesthesia and surgical steps were consistent with the tMCAO cohort, except that no actual MCA occlusion was performed-specifically, the filament was not inserted into the middle cerebral artery. During surgery, body temperature was strictly maintained at 37 ± 0.5 °C using a heating pad. Post-operatively, mice received subcutaneous meloxicam (2 mg/kg, Metacam) immediately and daily for two consecutive days. To accommodate stroke-induced motor deficits, softened food and accessible water were placed directly on the cage floor. Animals that died during or immediately after surgery, those with subarachnoid hemorrhage were excluded from the final analysis. At the end of the experiment, mice were euthanized by intraperitoneal injection of an overdose of sodium pentobarbital (200 mg/kg). Death was confirmed by the absence of respiration, heartbeat, and corneal reflex.

### Adeno-associated virus injection and inhibitor

Consistent with our prior experimental protocols, adeno-associated virus (AAV) constructs-empty vector (AAV-empty) and short hairpin RNA targeting Syndecan-4 (AAV-sh_SDC4) at a titer of 1.04 × 10¹⁰ genome copies (gc)-were administered separately into the cerebral cortex and ipsilateral striatum of adult male mice 21 days prior to transient middle cerebral artery occlusion (tMCAO) surgery. For stereotaxic injections, a 10-µL Hamilton microsyringe (Hamilton Company, Reno, NV, USA) was used with coordinates relative to bregma: mediolateral (ML) + 2.1 mm, anteroposterior (AP) + 1.0 mm, and injection depths of 1.5 mm and 3.5 mm (for cortex and striatum, respectively). After each injection, the syringe was held in position for 3 min; following the final injection, it was retained for an additional 15 min to minimize reflux of the viral solution. Additionally, XAV-939-a small-molecule inhibitor of the Wnt/β-catenin signaling pathway (40 mg/kg body weight; Selleck Chemicals, Houston, TX, USA; Cat# S1180)-was delivered via intraperitoneal (i.p.) injection twice: 2 days before tMCAO and on the day of surgery immediately prior to occlusion induction^25^.

### Evaluation of neurological function

Neurological deficits were assessed in experimental mice prior to the induction of middle cerebral artery occlusion (MCAO), as well as at post-MCAO time points including 6 h,12 h,24 h,3 days, and7 days. To evaluate sensorimotor function across different experimental groups, we employed the modified neurological severity score (mNSS)^26^. This scoring tool ranged from0 (indicating no detectable neurological deficits) to18 (representing severe, widespread impairment), and was used to quantify the degree of neurological damage in the mice.

For assessing dynamic balance and motor coordination in experimental mice following stroke induction, we utilized the Ugo Basile Model47600 rota-rod apparatus (Milan, Italy), as described previously. To optimize test performance, a 3-day acclimation and training phase was implemented prior to transient middle cerebral artery occlusion (tMCAO) surgery. The apparatus was configured to ramp up rotational speed from 4 rotations per minute (rpm) to 40 rpm over a 5-minute (300-second) period. Each day of testing involved three consecutive trials, with a 15-minute rest interval between individual trials to minimize fatigue. The primary outcome measure recorded was the latency to fall from the rotating rod.

Neuromuscular function in the studied animals was evaluated through a grip strength test. Adaptive pre-training was commenced 72 h prior to MCAO induction, as reported elsewhere^27^. During the test, each mouse grasped the grip plate of a commercial strength meter (Yiyan Technology Development Co., Ltd., Shandong Province, China) using both forelimbs. The experimenter then applied a gentle backward pull to the mouse’s tail, documenting the maximum force exerted by the animal before it released the plate.

### Hematoxylin–Eosin (HE) staining

Briefly, animals were first perfused with 0.1 M phosphate-buffered saline (PBS), followed by perfusion with 4% paraformaldehyde (PFA). After brain extraction, cerebral tissues were immersion-fixed in 4% PFA, then subjected to sequential dehydration using a graded ethanol gradient (70% to 100%), clearing with xylene, and embedding in paraffin wax. Brain sections of the desired thickness were cut using a rotary microtome (Leica Biosystems, Germany) and mounted onto microscope slides. For histological assessment, deparaffinized sections underwent hematoxylin staining (1 min), three rinses with deionized water, followed by differentiation in 1% acidified ethanol for 30 s. This was followed by eosin counterstaining (50 s), then final dehydration using absolute ethanol. Coverslips were affixed using neutral balsam mountant. Morphological analysis was performed under bright-field microscopy (Olympus Corporation, Japan), with digital images captured at standardized magnifications.

### Nissl staining

First, we perfused mice with 0.1 M PBS, followed by 4% PFA perfusion. Isolated mouse brains were immersion-fixed in 4% PFA, then processed through a graded ethanol series for dehydration, cleared with xylene, and infiltrated with paraffin wax. Paraffin-embedded brains were sectioned transversely using a rotary microtome (Leica Biosystems, Germany). Dewaxed sections were stained with Nissl solution (comprising sodium tetraborate and toluidine blue) and incubated at room temperature for 5 min. Subsequently, sections were differentiated in 1% glacial acetic acid for 3 s, then rinsed thoroughly with distilled water. After rinsing, sections were dehydrated, cleared with xylene, and mounted under coverslips. Finally, images were captured using a bright-field microscope (Olympus Corporation, Japan).

### 2,3,5-Triphenyltetrazolium chloride (TTC) staining

The infarct volume was measured by TTC staining. The incubation of brain slices with 2% TTC (Millipore, Sigma) was performed at 37 °C for 25 min, and then the slices were fixed. The white areas are the cerebral infarction. Stained slices were observed and then the images were analyzed by ImageJ software. As previously reported^28^, the infarct volume was obtained by integrating the infarct areas in all sections, and it is expressed as a percentage of infarct area.

### Terminal deoxynucleotidyl transferase-dUTP nick end labeling (TUNEL) assay

TUNEL assays were conducted to assess neuronal cell death in mouse brain tissues. First, paraffin-embedded sections were dewaxed twice in xylene (10 min per incubation) to ensure complete paraffin removal. Subsequently, sections were treated with dropwise Proteinase K (20 µg/mL; Beyotime) for 25 min at room temperature, followed by incubation with Enhanced Peroxidase Blocking Buffer (Beyotime) to minimize non-specific background staining. Cell death was visualized using the Colorimetric TUNEL Kit (Beyotime), strictly adhering to the manufacturer’s protocol. Finally, stained sections were examined under a fluorescence microscope (model BX60; Olympus Corporation, Tokyo, Japan), with digital images captured at consistent magnification and exposure settings.

### Assessment of brain water content

Brain water content was assessed via the dry-wet weight method, as detailed in a prior study^29^. First, mice were euthanized. Following euthanasia, brains were rapidly harvested, and cerebral hemispheres were dissected and separated. Wet weights of individual hemispheres were immediately measured using a precision analytical balance. For dry weight determination, hemispheres were placed in a drying oven set to 105 °C for 24 h until constant weight was achieved. Brain water content was calculated using the formula: [(Wet weight – Dry weight)/Wet weight] × 100%. Results are presented as the percentage of water content in cerebral hemispheres.

### Primary culture of microglia

Microglial cultures were established using protocols described previously^30^. Cortical tissues were enzymatically dissociated, and the resulting cell suspension was seeded into culture flasks. Cells were incubated in a humidified environment (37 °C, 5% CO₂) in growth medium composed of Minimum Essential Medium (MEM; Fisher Scientific) supplemented with 10% fetal bovine serum (FBS; Fisher Scientific) and 1% penicillin-streptomycin (antibiotic-antimycotic solution; Fisher Scientific).All media components and reagents were sterile-filtered before application to ensure sterility. After 12–14 days of incubation, mature microglia were isolated from mixed glial cultures via orbital shaking at high speed. Isolated cells were resuspended in MEM containing 3% FBS and then transferred to cell culture plates for subsequent experiments.

### Lentivirus transfection and inhibitor

As per the instructions from the manufacturer, the cortical microglia were transfected with GFP-containing LV-vector, LV-sh_SDC4, respectively. Three days after the transfection, the cortical microglia underwent diverse treatments and were then subjected to distinct experimental procedures. XAV-939 (10 µM) was added into the microglia medium immediately after OGD^31^.

### Quantitative reverse transcription-polymerase chain reaction (qRT-PCR)

mRNA expression levels were assessed using quantitative reverse transcription polymerase chain reaction (qRT-PCR). Total RNA was isolated from samples using TRIzol reagent (Thermo Fisher Scientific, Waltham, MA, USA). Following RNA isolation, we quantified RNA concentration via a NanoDrop 2000 spectrophotometer (Thermo Fisher Scientific) and employed the iScript Reverse Transcription Supermix (Bio-Rad Laboratories) for complementary DNA (cDNA) synthesis. Amplification was performed using SYBR^®^Premix Ex Taq™II (Takara, Japan) for annealing and extension^32^. The primer sequences used in qRT-PCR were as follows: SDC4 (forward, 5’-AACTGAGGTCTTGGCAGCTC-3’ ; reverse, 5’-TACACCAGCAGCAGGATCAG-3’). Claudin-5 (forward, 5′-ATCGGTGAAGTAGGCACCAA-3′; reverse, 5′-CTGCCCTTTCAGGTTAGCAG-3′); Occludin (forward, 5′-TGAATGGCAAGCGATCATAC-3′; reverse, 5′-TGCCTGAAGTCATCCACACT-3′). ZO-1 (forward, 5′-ATTTACCCGTCAGCCCTTCT-3′; reverse, 5′-TCGCAAACCCACACTATCTC-3′); β-actin (forward, 5′-AGCCATGTACGTAGCCATCC-3′; reverse, 5′- ACCCTCATAGATGGGCACAG-3′).

### Establishment of a microglia oxygen and glucose deprivation/reoxygenation (OGD/R) model

Briefly, we first replaced the standard growth medium with glucose-free DMEM (Gibco) and induced cellular injury by incubating cells under hypoxic conditions (95% N₂, 5% CO₂) for 2 h^33^. After OGD exposure, cells were incubated in standard growth medium and maintained in a normoxic incubator (5% CO₂, 95% air) for 4 h to promote recovery. Negative control cells were not subjected to OGD exposure. For genetic manipulation, we performed cell transfection using LV-sh_SDC4 according to the manufacturer’s protocol.

### Mitochondrial staining

Mitochondrial morphology was assessed using MitoTracker Red CMXRos (Invitrogen) per the manufacturer’s protocol^34^. Briefly, primary microglia seeded onto glass coverslips were rinsed twice with warm phosphate-buffered saline (PBS). Cells were then co-stained with 4’,6-diamidino-2-phenylindole (DAPI) for nuclear labeling and 100 nM MitoTracker Red CMXRos for mitochondrial visualization, with incubation at 37 °C for 20 min in the dark to preserve fluorophore integrity. Subsequently, the cells were preserved using formalin to facilitate morphological visualization. Observations of the images were conducted with a confocal microscope (LSM 750, Zeiss, Göttingen).

### Mitochondrial membrane potential measurement (ΔΨm)

ΔΨm alterations were assessed using a JC-1 assay kit (Beyotime Institute of Biotechnology, Shanghai, China; Catalog No. C2003S). Briefly, primary cortical microglial cells cultured in 25 cm² flasks (cell density: 3 × 10⁶ per flask) were collected and spun down at 1000 rpm for 5 min. The pellet obtained was reconstituted in 2 mL of the working reagent and incubated at 37 °C for 20 min in dark conditions, then centrifuged again at 1000 rpm for 5 min. Next, the pellet was washed with ice-cold buffer, and samples were assessed via a BD Accuri C6 Plus flow cytometer (BD Biosciences, San Jose, CA, USA). Flow cytometry data were analyzed with FlowJo software v10.8.1 (BD Biosciences, San Jose, CA, USA), with results reported as the ratio of red-to-green fluorescence intensity^35^.

### Western blot

Briefly, protein was applied to electrophoresis, blocking, and incubation with the primary antibodies: SDC4(1:1000, Cat# sc-12766; Santa Cruz 5G9), β-actin (1:5000, Cat# YT0099; Immunoway), Cyt c (1:1000, Cat: GB11080; Servicebio), AIF (1:1000, Cat# A19536; ABclonal), Tomm20 (1:1000, Cat# ab56783; Abcam), APC (1:1000, Cat# A17912; ABclonal), Axin-1 (1:1000, Cat# A16019; ABclonal), β-Catenin (1:1000, Cat# 8480; Cell Signaling Technology). After performing three washes, HRP-conjugated goat antibodies that are specific to rabbit or mouse IgG (both sourced from Millipore) were applied at room temperature for a duration of 60 min. Post incubation, visual imaging of the bands was done using a FluorChem M system (ProteinSimple, San Jose).

### Reactive oxygen species (ROS) quantifications

ROS levels in microglial cells were assessed using dihydroethidium (DHE) fluorescent dye (Beyotime Biotechnology, Shanghai, China; Cat# S0063). First, cells were seeded into six-well plates at a density of 3 × 10⁵ cells per well. They were then incubated with 3 µM DHE at 37 °C for 30 min in the dark (to minimize fluorophore degradation) and subsequently rinsed three times with ice-cold PBS. After staining, cells were visualized under a Leica DMI8 inverted fluorescence microscope. Finally, the fluorescence intensity of DHE signals was processed and quantified using ImageJ software (version 1.53t, National Institutes of Health, USA)^36^.

### Fluorescence-activated cell sorting (FACS) analysis

Collect cell suspensions from experiments, treat them with 1% bovine serum albumin, and wash and resuspend in PBS. Incubate the samples with antibodies (1 µL/tube) on ice in the dark for 30 min. After washing again, analyze the cells using FACS Canto II flow cytometry (BD Biosciences, USA). For intracellular antigen staining, add 0.1% cell activation cocktail (containing brefeldin A) 4 h prior to cell collection. Surface antigen staining of cells was performed, followed by fixation using the Cyto-Fast™ Fix/Perm Buffer Set (BioLegend, USA), permeabilization, and incubation on ice in the dark for 30 min. Antibodies for FACS: PerCP-Cy5.5 CD11b, APC-Cy7 CD45. All staining procedures were performed in the dark. Flow cytometry and data analysis were conducted using BD FACSCanto II (BD Bioscience, San Jose, CA, USA) and FlowJo software.

### Immunofluorescent staining

Following a modified protocol from prior research, immunofluorescence staining was carried out on tissue section^37^. First, sections were blocked in 5% bovine serum albumin (BSA) blocking buffer for 1 h at room temperature prior to overnight incubation (4 °C) with primary antibodies: Occludin (1:500, Cat# AB216327; Abcam), Claudin-5 (1:200, Cat#343214; ZenBio), CD31 (1:100, Cat#28083-1-AP; Proteintech), and cytochrome c (Cyt c;1:100, Cat#GB11080; ServiceBio). Next, sections were incubated with species-matched secondary antibodies for 2 h at room temperature: CoraLite488-conjugated Goat Anti-Rabbit IgG (Proteintech) and Alexa Fluor594-conjugated Goat Anti-Mouse IgG (Abcam). Subsequently, cell nuclei were counterstained with DAPI (Cat#C3606; Beyotime, Shanghai, China) for 15 min. Finally, stained sections were visualized using a Zeiss LSM750 confocal microscope (Zeiss, Göttingen, Germany).

### Co-immunoprecipitation assay (Co-IP)

We conducted co-immunoprecipitation (co-IP) assays by homogenizing cell pellets in IP lysis buffer supplemented with protease inhibitors (Progena, Madison), phosphatase inhibitors (Roche), and 5 mM N-ethylmaleimide (Sigma-Aldrich). Cell lysates were incubated with target-specific primary antibodies for 2 h at 4 °C under constant gentle rotation to facilitate antigen-antibody binding. Next, Protein A/G agarose beads (Calbiochem) were added to the mixture and incubated for an additional 4 h at 4 °C to capture immune complexes. Following three sequential washes with cold IP buffer to eliminate non-specific interactions, immunoprecipitated proteins were eluted using 2× Laemmli buffer and separated via SDS-PAGE prior to immunoblotting analysis. For immunoblotting detection, primary antibodies against SDC4 (Santa Cruz Biotechnology) and Dvl (Santa Cruz Biotechnology) were utilized to identify target antigens.

### Molecular dynamics (MD) trajectories analysis and molecular docking

This study retrieved the corresponding structures from Uniprot. The above structures are all derived from AlphaFold2 modeling results^38^. Based on the modeling results, the regions at the N-terminal of the protein with high B-Factor flexibility and low precision were truncated.

Select HDock for docking, and use PyMOL for visualization (it is open-source software and does not require citation)^39^. Perform analysis using LigPlot+^40^.

Select the docked result conformation as the initial conformation for dynamics. Choose Gromacs2019.6 as the dynamics simulation software, with amber14sb as the protein force field^41^. Apply the TIP3P water model to the complex system to add TIP3P water molecules, construct a water box, and add sodium ions to balance the system. The Coulomb force cutoff distance and van der Waals radius cutoff distance were both set to 1.4 nm. The system was then equilibrated using the canonical ensemble (NVT) and the isothermal-isobaric ensemble (NPT). The Particle-mesh Ewald (PME) method was employed with a cutoff value set to 1.2 nm. The non-bonded interaction cutoff was set to 10 Å. The V-rescale temperature coupling method was used to maintain the simulation temperature at 300 K, and the Berendsen method was employed to control the pressure at 1 bar. At 300 K, 30 ps NVT and NPT equilibrium simulations were performed respectively; finally, a 100 ns production MD simulation was conducted on the protein-ligand complex system. Root mean square deviation (RMSD) was used to observe the local site conformational changes of the system during the simulation (the fluctuation cutoff was set to 0.2). Radius of gyration (Rg) was employed to evaluate the compactness of the system structure. Root mean square fluctuation (RMSF) could be used to observe the local site conformational changes of the system during the simulation. SASA (Solvent Accessible Surface Area) is used to observe the size of the solvent accessible surface area of the complex during the simulation process.

### Statistical analysis

Statistical evaluations and figure generation were conducted via GraphPad Prism 8.4.3 (GraphPad Software, San Diego, CA), with quantitative values reported as mean ± standard error mean (SEM). The normal distribution of the data and the homogeneity of variance were verified using the Shapiro-Wilk and Brown-Forsythe tests, respectively. For data with normality and equal variance, one-way ANOVA with Bonferroni’s post hoc test was employed. Behavioral scores were analyzed utilizing a two-way repeated-measures ANOVA alongside Bonferroni’s post hoc analysis to assess treatment efficacies. Differences were considered statistically significant when *P* < 0.05. All behavioral tests, infarct volume measurements, and histological quantifications were performed by investigators blinded to the experimental groups.

## Results

### Single-cell RNA sequencing analysis identifies cell lineages at different stages of MCAO

**Fig. 1:**
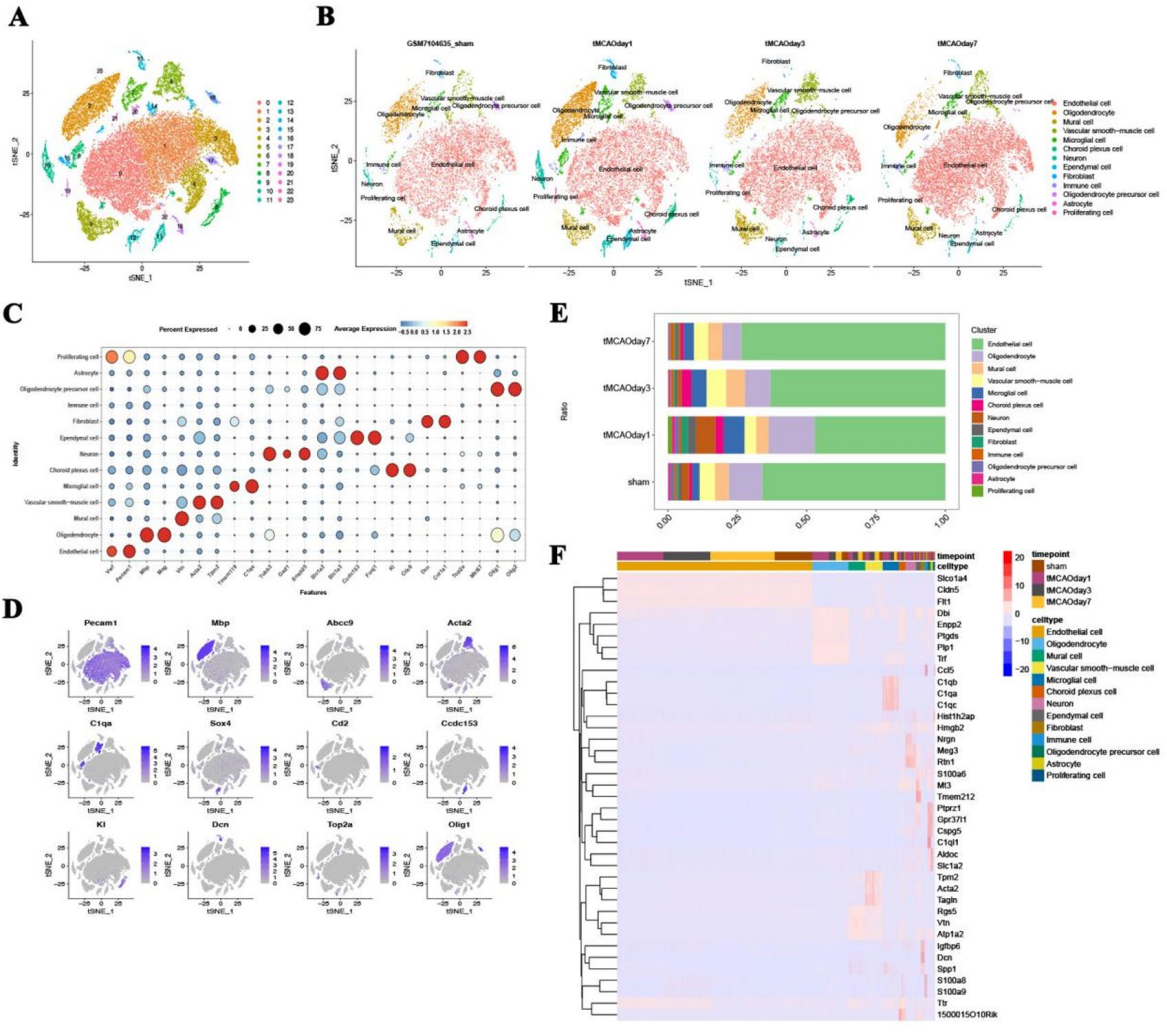
Identification of cell types. (**A**) Single-cell RNA-sequencing determines major cell clusters. (**B**) t-SNE plot showing cell types in sham and MCAO (1-d, 3-d, and 7-d post-ischemic stroke) mouse brains. Colors highlight 13 transcriptionally distinct cell types. Each colored dot corresponds to an individual cell. (**C**) Dot plot showing the expression of previously reported marker genes in 13 cell types. (**D**) t-SNE visualizations emphasize the primary cell types based on marker gene expression. (**E**) Proportional histogram depicting the proportion alterations of 13 transcriptionally distinct cell types at the acute stage of ischemic stroke (Sham, MCAO-1d, MCAO-3d, and MCAO-7d). (**F**) Heatmap showing the expression of the DEGs of each cell type.

This study was conducted based on the GSM7104632 dataset, analyzing a total of 4 tissue samples, including 1 sham sample and 3 tMCAO samples, with the tMCAO samples categorized into day1, day3, and day7. The tSNE algorithm was employed for visual dimensionality reduction, identifying 24 cell clusters (Fig. 1A). The 24 subtypes were biologically annotated through known cell markers, and identified as Endothelial cell, Oligodendrocyte, Mural cell, Vascular smooth-muscle cell, Microglial cell, Choroid plexus cell, Neuron, Ependymal cell, Fibroblast, Immune cell, Oligodendrocyte precursor cell, Astrocyte, and Proliferating cell (Fig. 1B). Next, we present the characteristic marker genes of each cell type (Fig. 1C,D) and the proportion of each cell type in the four tissue samples (Fig. 1E). Analysis of cell proportions revealed a significant increase in the proportion of microglia and astrocytes one day after ischemic injury, which then decreased by day 7 post-MCAO. We compared the differentially expressed genes between each cell type, obtained a list of significantly different genes, and displayed them using a heatmap (Fig. 1F). In summary, our results demonstrate significant changes in the major cell types between the normal group and the MCAO group.

### Anatomy and clustering of microglia in cortical MCAO samples

**Fig. 2:**
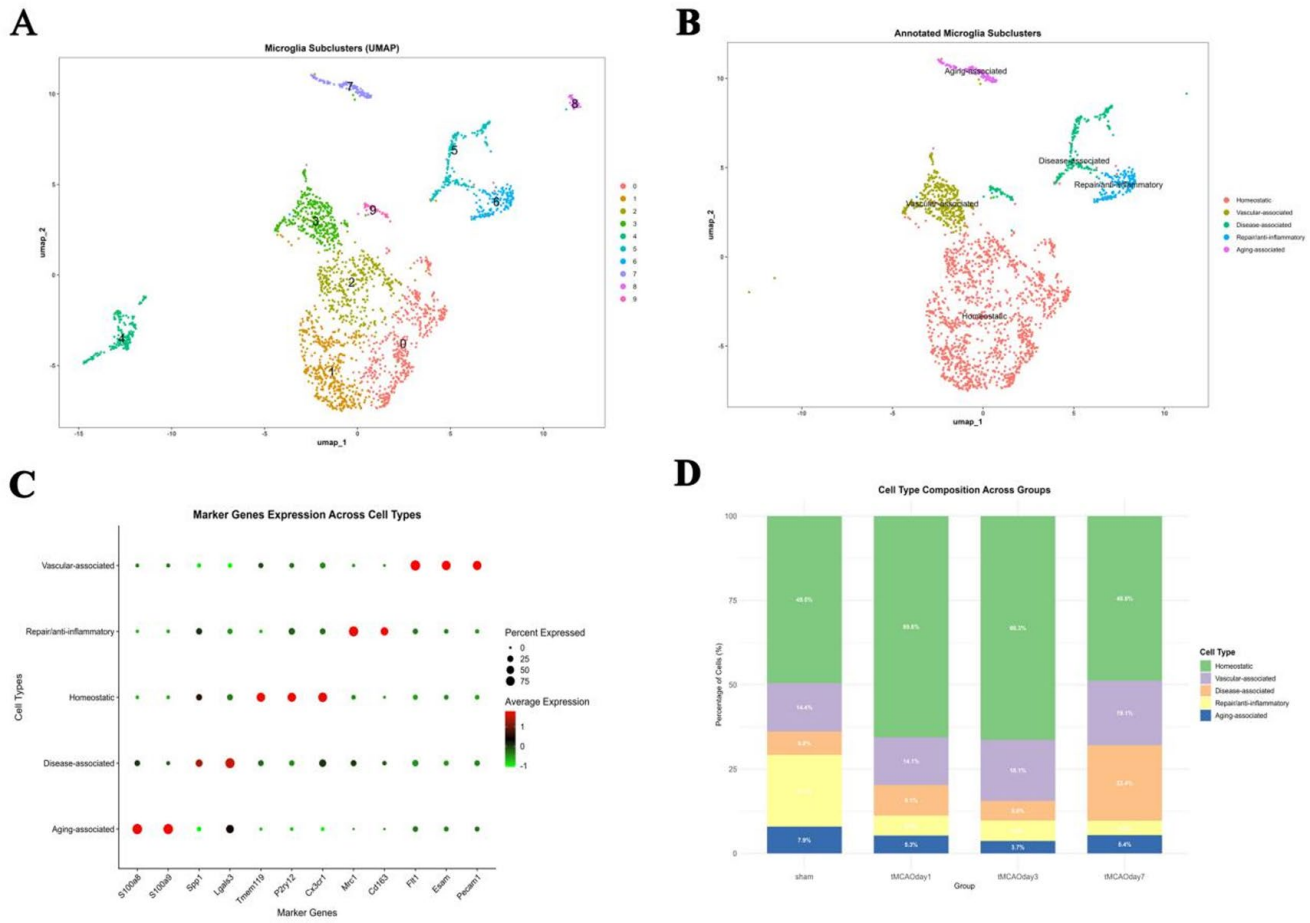
Molecular mapping of phenotypic heterogeneity among microglial subpopulations. (**A**) t-SNE plot showing the distribution of microglial clusters. Each colored dot corresponds to a microglial subpopulation. (**B**) t-SNE plot showing the distribution of defined microglial subtypes. (**C**) Dot plot showing the expression of conserved marker genes among the five microglial subpopulations. (**D**) Bar graph showing the proportion of each defined microglial subtype between the control group and the 1, 3, and 7 day MCAO groups.

To gain deeper insights into the phenotypic diversity of microglia following middle cerebral artery occlusion (MCAO), we conducted additional clustering analyses focused on these cell populations. Specifically, Uniform Manifold Approximation and Projection (UMAP)-based clustering revealed 10 discrete microglial cell clusters (Fig. 2A). Analysis using the FindAllMarkers function revealed that the highly expressed genes in clusters 4 and 8 are not microglial-expressed. Therefore, clusters 4 and 8 were removed during cell annotation. The final annotation results are shown in Fig. 2B, revealing five microglial subpopulations: homeostatic, vasculature-associated, disease-associated, repair/anti-inflammatory, and aging-associated. Next, we present the characteristic marker genes of each subpopulation (Fig. 2C). Significant differences in the proportions of these subpopulations were observed among the four groups. Specifically, in the control group, the proportions of homeostatic, disease-associated, repair/anti-inflammatory, and aging-associated microglia were 49.5%, 6.9%, 21.3%, and 7.9%, respectively. However, in the 7-day MCAO group, the proportions of homeostatic, and aging-associated microglia decreased to 48.8%, and 5.4%, respectively. The proportions of disease-associated and vascular-associated microglia increased to 22.4% and 19.1%, respectively, after 7 days of MCAO (Fig. 2D). In summary, our findings demonstrate that several distinct cellular states within microglia evolve over time following MCAO.

### Analysis of SDC4 expression in microglial subpopulations

**Fig. 3:**
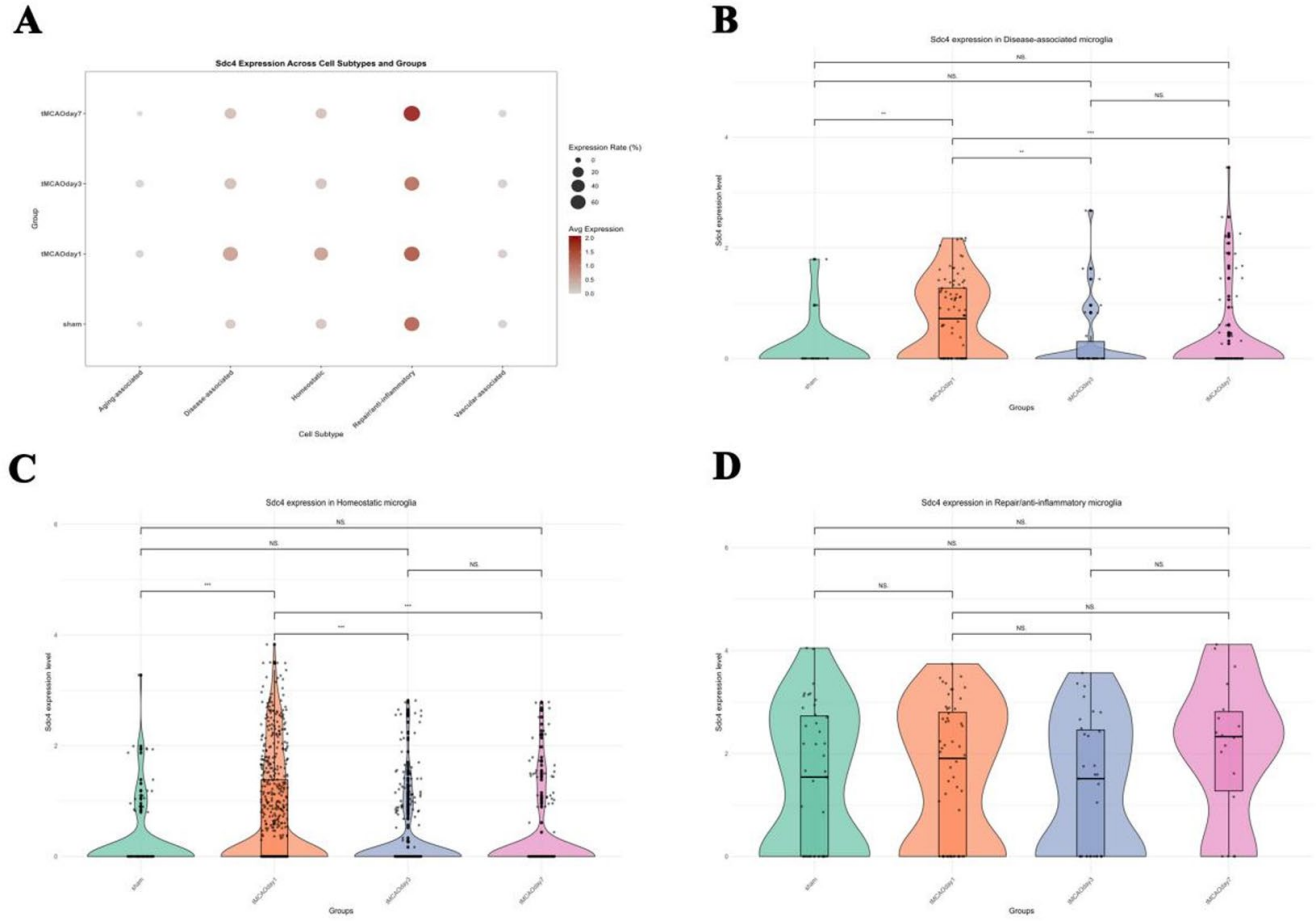
Analysis of SDC4 Expression in Microglial Subpopulations. (**A**) Dot plot shows the expression of SDC4 in five microglial subsets. (**B**) Violin plot shows the expression of SDC4 in disease-associated microglial cells at 1, 3, and 7 day MCAO groups. (**C**) Violin plotshows the expression of SDC4 in homeostatic microglial cells at 1, 3, and 7 day MCAO groups. (**D**) Violin plot shows the expression of SDC4 in repair/anti-inflammatory microglial cells at 1, 3, and 7 day MCAO groups.

To further investigate significant differences in SDC4 expression among the groups, we analyzed expression differences using the Wilcoxon test. The expression of SDC4 across different cell subpopulations is illustrated in Fig. 3A. As shown, SDC4 is primarily expressed in the disease-associated, homeostatic, and repair/anti-inflammatory subpopulations. Specifically, the expression of SDC4 in disease-associated microglia at various time points is depicted. Notably, SDC4 levels were significantly elevated 1 day after MCAO compared to the sham group (Fig. 3B). Figure 3C presents the expression of SDC4 in homeostatic microglia over different time points, revealing a similar trend of significant elevation 1 day post-MCAO compared to the sham group. Figure 3D depicts the expression of SDC4 in repair/anti-inflammatory microglia at various time points. Collectively, these results suggest that SDC4 expression levels initially increase and subsequently decrease at different time points following MCAO.

### Microglial transcriptional trajectories

**Fig. 4:**
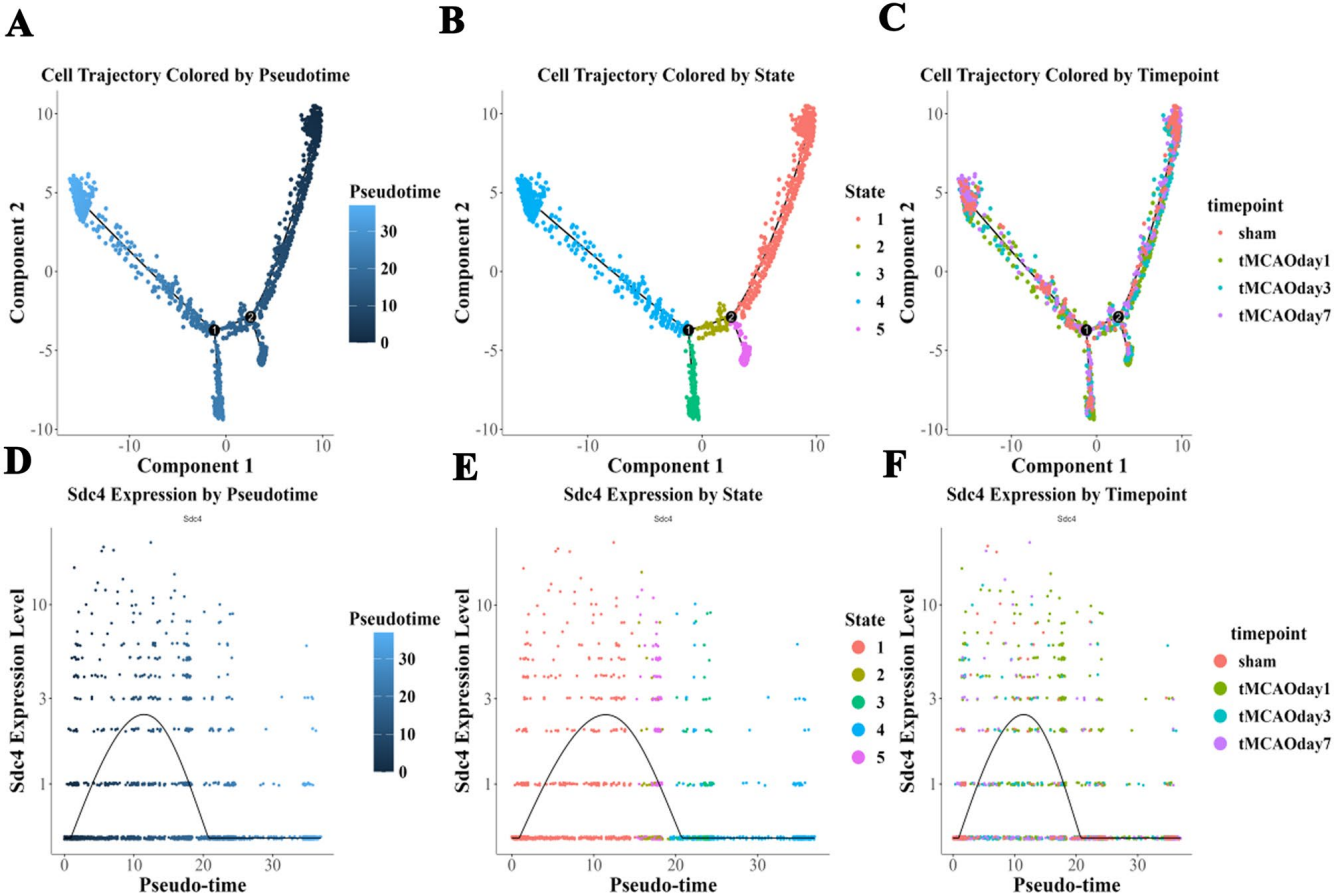
Trajectory analysis of SDC4 in microglia. (**A**–**C**) Trajectory plots show the cellular transition state of these cell types. (**D**–**F**) SDC4 expression changes over time.

To investigate the expression of SDC4 at various cellular stages, pseudo-time analysis was performed using the R package monocle (2.36.0). Cell trajectory diagrams, color-coded by pseudo-time, show the cellular transition state trajectories of microglial subpopulations in principal component space as pseudo-time progresses (Fig. 4A). Cell trajectory diagrams, color-coded by state, show the distribution of cells in each state along the cellular transition state trajectory, reflecting the evolutionary relationship between cell states. Five distinct cellular transition states and two independent branch points were ultimately identified. Homeostatic microglia appear at the beginning of the trajectory, repair/anti-inflammatory, disease-associated and aging-associated microglia are primarily located in the center, and vascular-associated microglia appear at the ends of the trajectory (Fig. 4B). A color-coded cell trajectory diagram, grouped by time point, shows the position of microglia in different groups along the cellular transition state trajectory. The diagram demonstrates that cells at different stages of cellular transition exist at each time point (Fig. 4C). Next, based on pseudo-time series analysis, a scatter plot of SDC4 gene expression was plotted over time. The results showed that SDC4 expression initially increased, then decreased, reaching near-zero expression in the late stages of cellular transition (Fig. 4D). Next, we plotted the pseudo-time evolution of SDC4 expression by cell state, with different colors representing different states. Combined with fitted curves, the dynamic differences in gene expression within each state were demonstrated. The results showed that SDC4 expression levels in homeostatic and disease-associated microglia initially increased and then decreased (Fig. 4E). Finally, we present a pseudo-timeline plot of SDC4 expression grouped by time point, with different colors representing each group. This plot illustrates the temporal trends of SDC4 expression within each group. A color-coded cell trajectory plot, categorized by time point, shows the detail of the cellular transition state trajectory at different time points. As shown, sham, tMCAO day 3, and tMCAO day 7 exhibit similar cell cellular transition state trajectories, but tMCAO day 1 differs (Fig. 4F). Overall, these findings suggest that SDC4 expression showed a transient increase during the early phase after MCAO and subsequently declined during later stages of disease progression.

### SDC4 exhibits high expression in microglia during tMCAO in mice

**Fig. 5:**
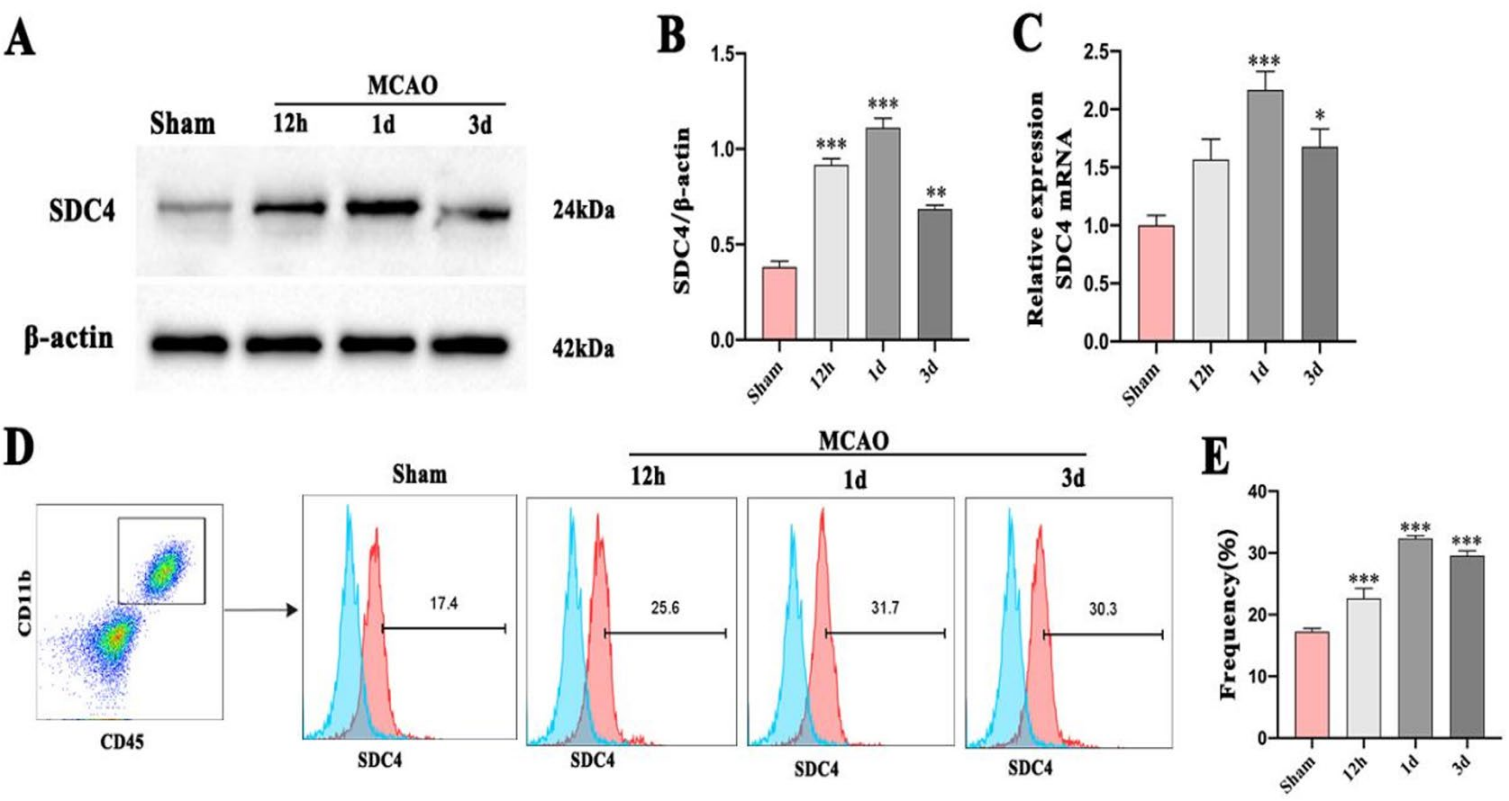
Upregulation of SDC4 in cerebral microglia cells in ischemia-reperfusion mouse model. (**A**, **B**) The protein expression and Western Blot analysis of SDC4 in the penumbra region of different groups; *n* = 3. Data are expressed as mean ± SEM. **P < 0.01, ***P < 0.001, as compared with the Sham group by one-way analysis of ANOVA with Bonferroni’s post hoc test. (**C**) qPCR analysis of SDC4 in the penumbra region of different groups; *n* = 4. Data are expressed as mean ± SEM.*P < 0.05, ***P < 0.001, as compared with the Sham group by one-way analysis of ANOVA with Bonferroni’s post hoc test. (**D**, **E**) FACS analysis of SDC4 expression in microglia after tMCAO; *n* = 3. Data are expressed as mean ± SEM. ***P < 0.001, as compared with the Sham group by one-way analysis of ANOVA with Bonferroni’s post hoc test.

To investigate the potential involvement of SDC4 in microglial dysregulation during ischemic stroke, we assessed SDC4 expression from a mouse model of MCAO. Western blot analysis indicated a marked elevation in SDC4 protein levels in MCAO mice relative to sham-operated controls (Fig. 5A,B). Consistent with this observation, qRT-PCR data demonstrated a significant upregulation of SDC4 mRNA expression following MCAO (Fig. 5C). Further characterization using flow cytometry revealed dynamic alterations in SDC4 levels within microglia over time (Fig. 5D,E). Collectively, these results indicate that SDC4 is substantially upregulated in the peri-infarct region after ischemic stroke and imply a possible association between SDC4 and the pathophysiology of cerebral ischemia.

### SDC4 interference attenuates neurological deficits and infarct volume in mice after I/R

**Fig. 6:**
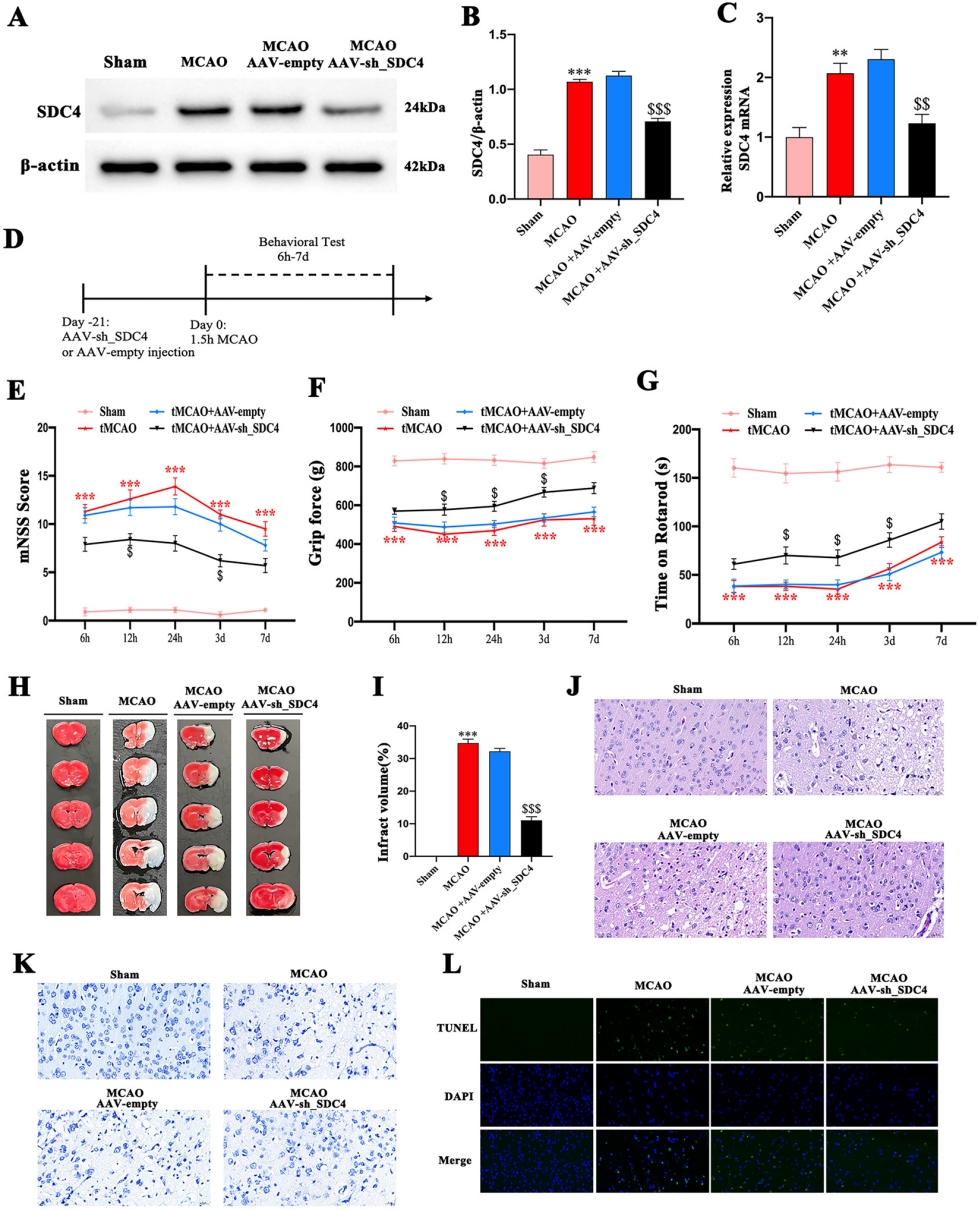
The effect of SDC4 interference on the neurological function, infarct volume in mice after MCAO. A, B Representative immunoblots and Western Blot analysis of SDC4 in the penumbra region of different groups; *n* = 3. Data are expressed as mean ± SEM. and analyzed by one-way analysis of ANOVA with Bonferroni’s post hoc test. ***p < 0.001, as compared with the Sham group; ^$$$^*p* < 0.001, as compared with the MCAO + AAV-empty group. C qRT-PCR analysis of SDC4 in the penumbra region of different groups. *n* = 4. Data are expressed as mean ± SEM. and analyzed by one-way analysis of ANOVA with Bonferroni’s post hoc test. **p < 0.01, as compared with the Sham group; ^$$^*p* < 0.01, as compared with the MCAO + AAV-empty group. D Schematic diagram of the experimental timeline. E mNSS score, F grip strength test, G rotarod test; *n* = 10. Data are expressed as mean ± SEM. and analyzed by repeated-measures analysis of variance (ANOVA) followed by Bonferroni’s multiple comparisons post-test. ***p < 0.001, as compared with the Sham group; ^$^*p* < 0.05, as compared with the MCAO + AAV-empty group. H, I TTC representative images and quantification of the infarct volume; *n* = 3. Data are expressed as mean ± SEM. and analyzed by one-way analysis of ANOVA with Bonferroni’s post hoc test. ***p < 0.001, as compared with the Sham group; ^$$$^*p* < 0.001, as compared with the MCAO + AAV-empty group. J-L Representative images of different groups by HE, Nissl and TUNEL staining at 1 day after MCAO; Scale bar = 20 μm.

The impact of SDC4 expression levels on focal cerebral ischemic injury was assessed via targeted injection of AAV-sh_SDC4 into the cerebral cortex and striatum of mice. Western blotting and qRT-PCR assays confirmed that this targeted delivery led to a significant reduction in SDC4 expression (Figs. 6A-C). Mice received a stereotaxic injection of AAV-sh_SDC4 or AAV-empty 21 days prior to the induction of tMCAO. The tMCAO surgery was performed on Day 0 (90 min of ischemia followed by reperfusion). Neurological behavioral tests were conducted from Hour 6 to Day 7 post-surgery (Figs. 6D). The influence of SDC4 on neurological and motor performance was evaluated at 6, 12, and 24 h, as well as 3 and 7 days post-tMCAO, using mNSS assessments, grip strength measurements, and rotarod tests, respectively. mNSS results revealed that SDC4 knockdown enhanced neurological function recovery post-MCAO relative to mice receiving empty AAV vectors (Fig. 6E). Similarly, grip strength was significantly higher in SDC4-interfered mice compared to the AAV-empty control group (Fig. 6F). In line with these findings, rotarod tests showed that SDC4 knockdown extended the duration mice remained on the rotating rod versus the AAV-empty-treated cohort (Fig. 6G). Additionally, TTC staining at 1 day post-MCAO demonstrated prominent cerebral infarction in both MCAO-only and AAV-empty-treated mice, a lesion that was significantly attenuated by SDC4 knockdown (Figs. 6H, I). Hematoxylin-eosin (HE) staining revealed that sham-operated mice exhibited well-arranged neurons with round or conical shapes, intact cellular structures, distinct nucleoli, and clear boundaries. In contrast, MCAO-only mice displayed marked signs of cellular damage: severe neuronal degeneration, disorganized tissue architecture, interstitial edema, swollen neurons with triangular or irregular morphologies, and nuclear pyknosis or fragmentation. Notably, SDC4 knockdown substantially mitigated these pathological changes (Fig. 6J). Nissl staining indicated that, relative to sham-operated controls, MCAO-only mice had fewer Nissl-positive cells and a significantly lower neuronal survival rate. Conversely, SDC4 knockdown significantly increased the count of Nissl-positive neurons and enhanced neuronal survival (Fig. 6K). Next, we performed Tunel staining, the proportion of apoptotic cells decreased significantly after SDC4 interference (Fig. 6L). Overall, these findings suggest that SDC4 interference attenuates sensorimotor deficits and tissue loss in mice after MCAO.

### SDC4 interference alleviates the damage to the mitochondrial morphology after OGD/R and retains the mitochondrial integrity

**Fig. 7:**
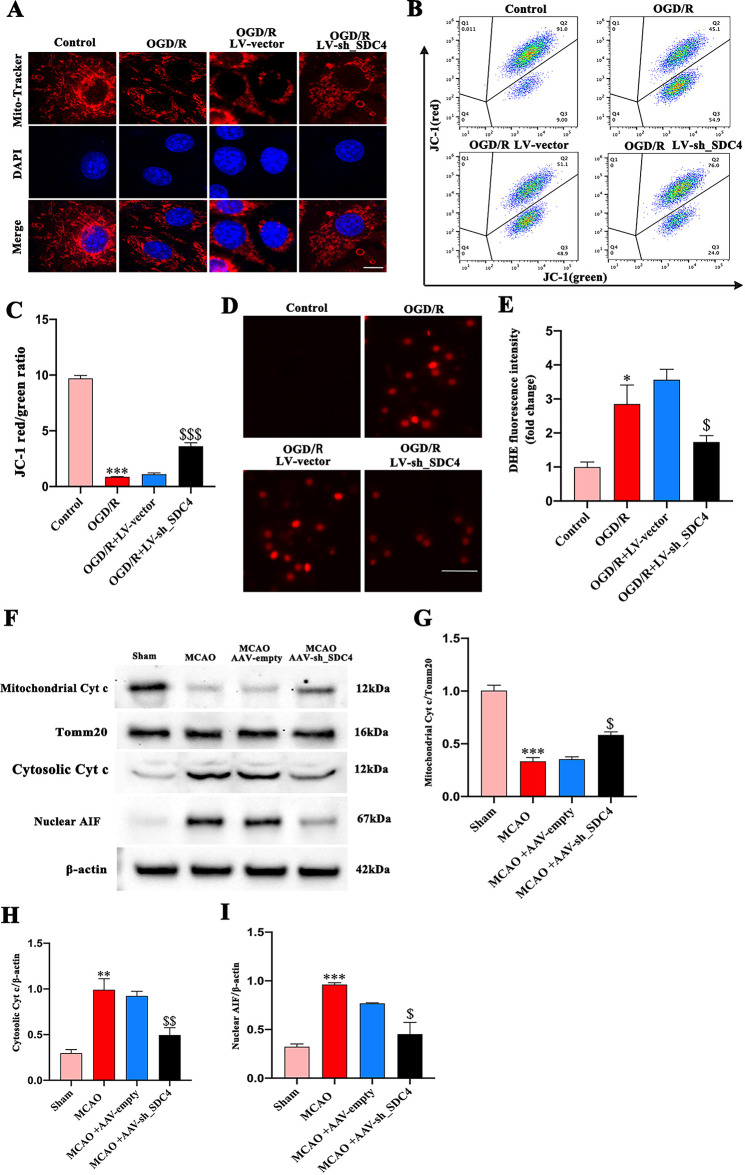
The retained mitochondrial integrity and alleviated mitochondrial morphology damage after OGD/R by SDC4 interference. A Representative fluorescence images. Scale bar: 20 μm. B, C JC-1 expression analyzed by flow cytometry and quantification of JC-1 expression; *n* = 3. Data are expressed as mean ± SEM. and analyzed by one-way analysis of ANOVA with Bonferroni’s post hoc test. ***p < 0.001, as compared with the Control group; ^**$$$**^*p* < 0.001, as compared with the OGD/R + LV-vector group. D, E DHE representative fluorescence images and quantification of fluorescence intensity in microglial cells; *n* = 3. Data are expressed as mean ± SEM. and analyzed by one-way analysis of ANOVA with Bonferroni’s post hoc test. *p < 0.05, as compared with the Control group; ^**$**^*p* < 0.05, as compared with the OGD/R + LV-vector group. Scale bar: 100 μm. F-I Immunoblot analysis and quantification of the mitochondrial Cyt c level, the cytosol Cyt c level, and the nuclear AIF level; *n* = 3. Data are expressed as mean ± SEM. and analyzed by one-way analysis of ANOVA with Bonferroni’s post hoc test. **p < 0.01, ***p < 0.001, as compared with the Sham group; ^**$**^*p* < 0.05, ^**$$**^*p* < 0.01, as compared with the MCAO + AAV-empty group.

Given that mitochondria’s integrity is rapidly compromised as an early response to ischemic injury-a critical pathological change-we next investigated whether SDC4 interference preserves mitochondrial structural stability. Mitochondria’s ultrastructure was evaluated via immunofluorescence staining at 12 h post-OGD/R. Relative to control cells, both OGD/R-exposed cells and LV-vector-transfected cells exhibited shortened mitochondrial length and fewer mitochondrial cristae. In contrast, SDC4 interference in OGD/R-exposed cells (vs. LV-vector controls) restored mitochondrial length and increased cristae density (Fig. 7A). Subsequently, JC-1 staining analysis revealed that OGD/R exacerbated mitochondrial membrane potential depolarization in microglial cells, as evidenced by increased green fluorescence (JC-1 monomers) and reduced red fluorescence (JC-1 aggregates). Notably, SDC4 interference significantly attenuated this depolarization in OGD/R-exposed microglia relative to LV-vector-transfected controls (Figs. 7B, C). DHE staining further showed that sh_SDC4 transfection reduced fluorescence intensity in OGD/R-exposed microglia compared to LV-vector-transfected counterparts (Figs. 7D, E). In vivo, WB assays demonstrated that MCAO-treated mice (and AAV-empty-injected controls) exhibited elevated cytosolic levels of AIF and Cyt c, alongside reduced mitochondrial Cyt c levels relative to sham-operated (untreated) mice. Conversely, SDC4 interference in MCAO mice significantly decreased cytosolic Cyt c levels and increased mitochondrial Cyt c levels relative to AAV-empty-injected controls (Figs. 7F-I). Collectively, these findings indicate that SDC4 interference effectively preserves mitochondrial integrity in the context of ischemic injury.

### SDC4 interference ameliorates I/R-induced BBB damage

**Fig. 8:**
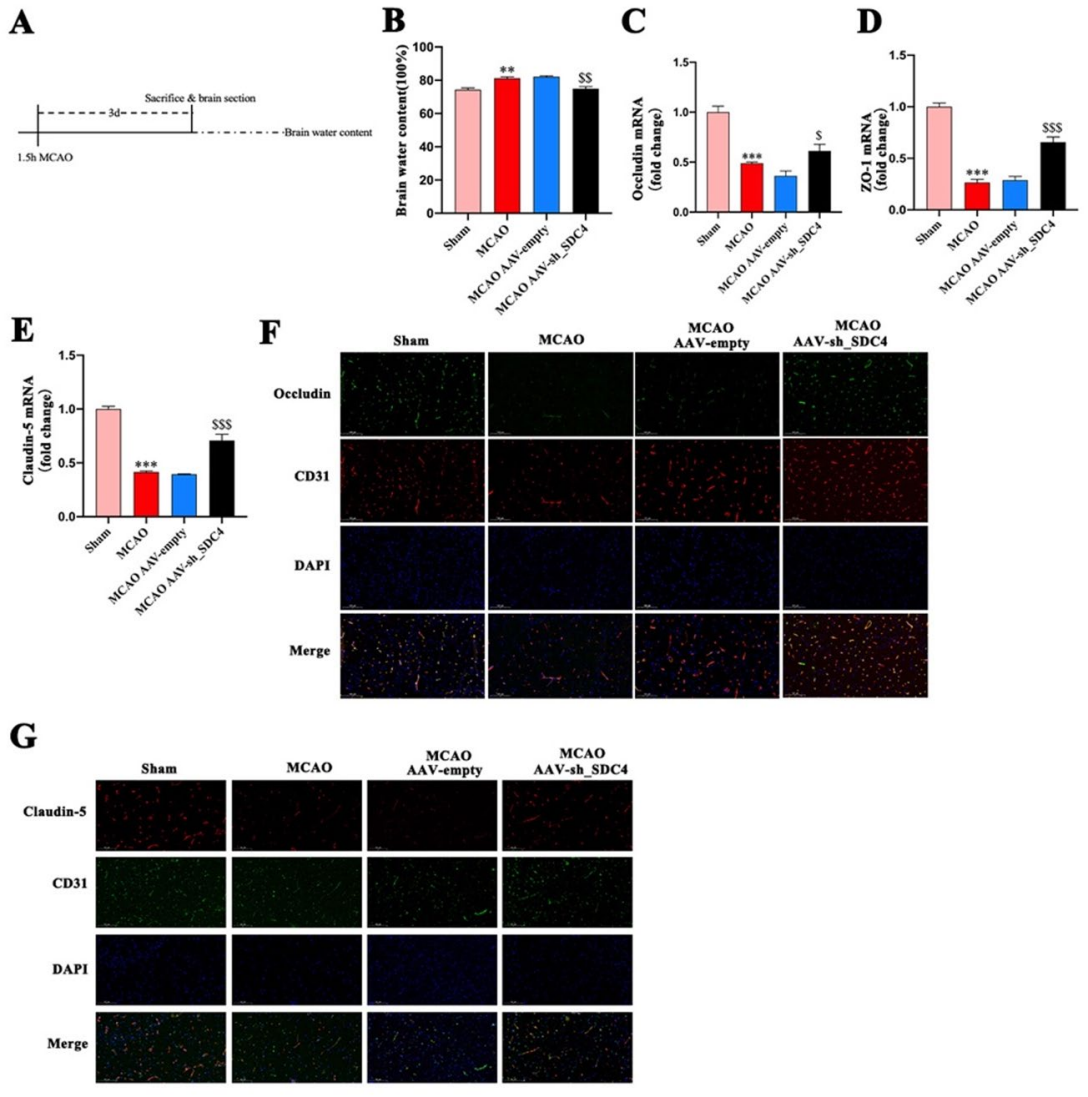
SDC4 interference decreases brain water content and BBB disruption in ischemic mice. A, B Water content was measured in dissected brains. *n* = 3. Data are expressed as mean ± SEM. and analyzed by one-way analysis of ANOVA with Bonferroni’s post hoc test. **p < 0.01, as compared with the Sham group; ^$$^*p* < 0.01, as compared with the MCAO + AAV-empty group. C-E qRT-PCR analysis of Occludin, ZO-1, Claudin-5 in the penumbra region of different groups; *n* = 4. Data are expressed as mean ± SEM. and analyzed by one-way analysis of ANOVA with Bonferroni’s post hoc test. ***p < 0.001, as compared with the Sham group; ^$^*p* < 0.05, ^$$$^*p* < 0.001, as compared with the MCAO + AAV-empty group. F Representative Immunofluorescence staining of occludin. Scale bar: 100 μm. G Representative Immunofluorescence staining of Claudin-5. Scale bar: 100 μm.

To evaluate BBB disruption after cerebral ischemia, We first performed brain water content test on samples from each group. Cerebral ischemia caused an increase in brain water content in both MCAO AAV-empty mice and MCAO mice (Fig. 8A, B). However, SDC4 interference significantly reduced the ischemia-reperfusion-induced water content. Next, we performed quantitative real-time PCR in the brain to detect BBB-related markers. Consistent with our hypotheses, mRNA levels of the tight junction proteins Claudin-5, occludin, and ZO-1 were markedly downregulated at 1 day post-focal cerebral ischemia. SDC4 interference effectively upregulated the expression of these BBB-associated tight junction proteins (Fig. 8C-E). Immunofluorescence staining further confirmed that I/R suppressed Claudin-5 and occludin protein expression, while SDC4 interference notably reversed these inhibitory effects (Fig. 8F-G). Collectively, these findings indicate that SDC4 interference preserves the structural and functional integrity of the BBB in MCAO-challenged mice.

### The simulations of molecular dynamics and the interaction between SDC4 and Dvl after OGD/R

**Fig. 9:**
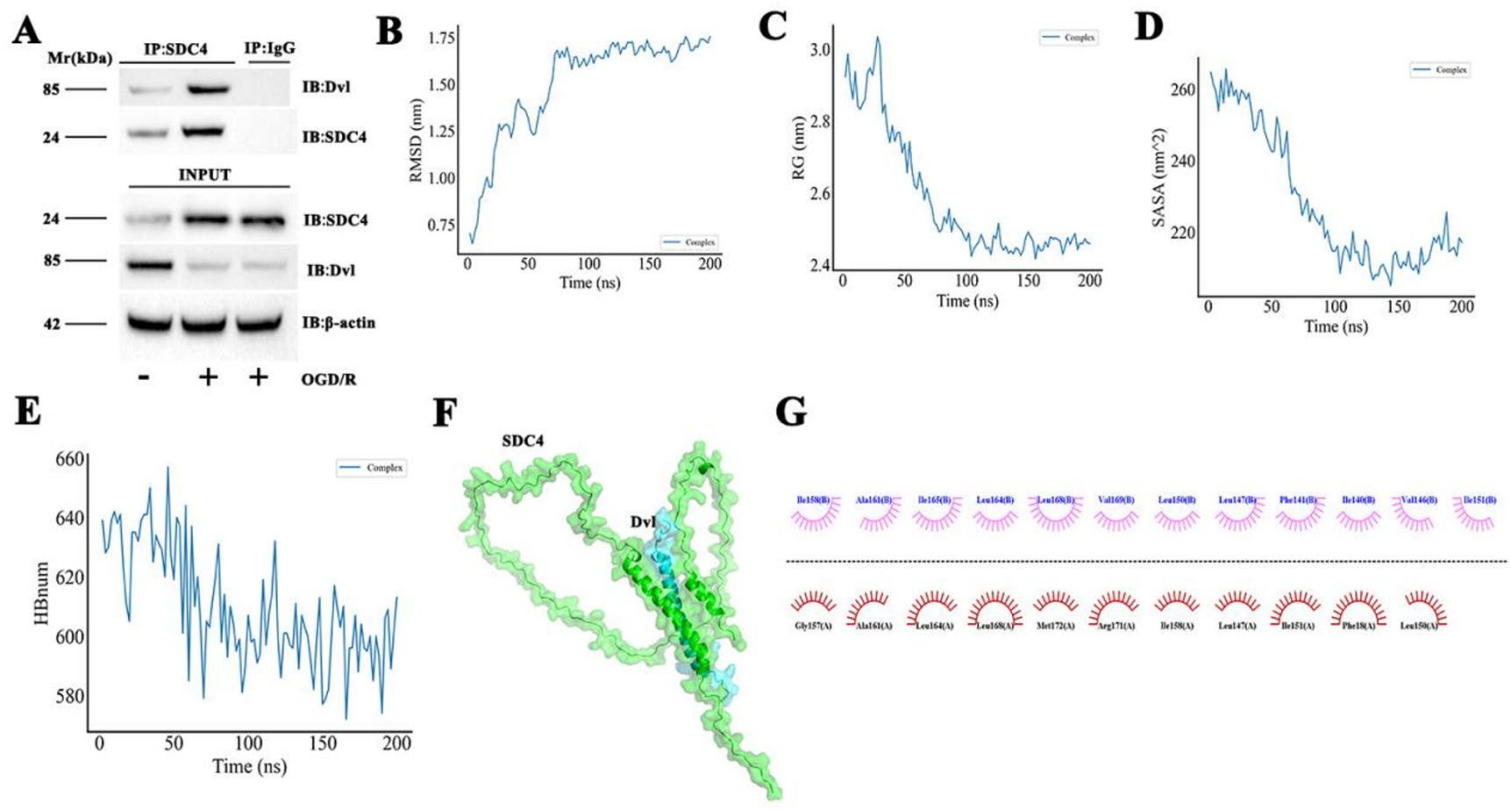
The analysis of MDS results. (**A**) The interaction of ectopically expressed SDC4 with Dvl was determined by Co-IP. (**B**) RMSD. (**C**) Rg. (**D**) SASA. (**E**) HB profiles of the SDC4 with Dvl. (**F**) Protein stable conformation display. Dvl is shown in blue, and SDC4 is shown in green. (**G**) The particular perspectives regarding the 2-D interaction of the ligand SDC4 with Dvl.

The CO-IP experiment demonstrated that the natural interaction between SDC4 and Dvl was significantly enhanced in cells following OGD/R (Fig. 9A). Molecular Dynamics Simulation (MDS) is a crucial method for investigating the stability and dynamic characteristics of complexes in aqueous solutions. The Root Mean Square Deviation (RMSD) serves as a metric to assess the stability of the system. All systems maintained stability during the initial simulation phase of 100–200 ns (Fig. 9B). The Radius of Gyration (Rg) is an important indicator for evaluating the compactness of the system’s structure. The fluctuations correspond precisely to the variations in RMSD, indicating that the system rapidly reaches equilibrium after achieving stability at 100 ns (Fig. 9C). SASA refers to the Solvent Accessible Surface Area of a protein. It was observed that the SASA of the protein steadily decreased from 0 to 200 ns, indicating favorable binding and gradual compaction of the protein (Fig. 9D). Hydrogen bond analysis further reflected the dynamic solvent interactions of the complex during the simulation (Fig. 9E). By analyzing the conformations during docking, the interaction relationship between the two proteins can be elucidated. Figure 9F reveals that the two proteins have extensive contact surfaces, and the α-helices of the two proteins form contacts. In the system of this study, it can be observed that due to the abundance of helical structures among proteins, these structures provide strong binding forces, making van der Waals forces the primary contact force, as exemplified by Gly157, Leu168, and Ile158 of SDC4; and Ala161, Val169, and Val146 of DVL (Fig. 9G). These interactions demonstrate the two proteins can bind together, with the primary binding force being hydrophobic interactions. During the binding process, the α-helix of the proteins serves as the main contact structure. The proteins gradually approach each other, with a reduction in surface area, leading to a more compact structure.

### SDC4 inhibits the Wnt/β-catenin signaling pathway in microglia following OGD/R

**Fig. 10:**
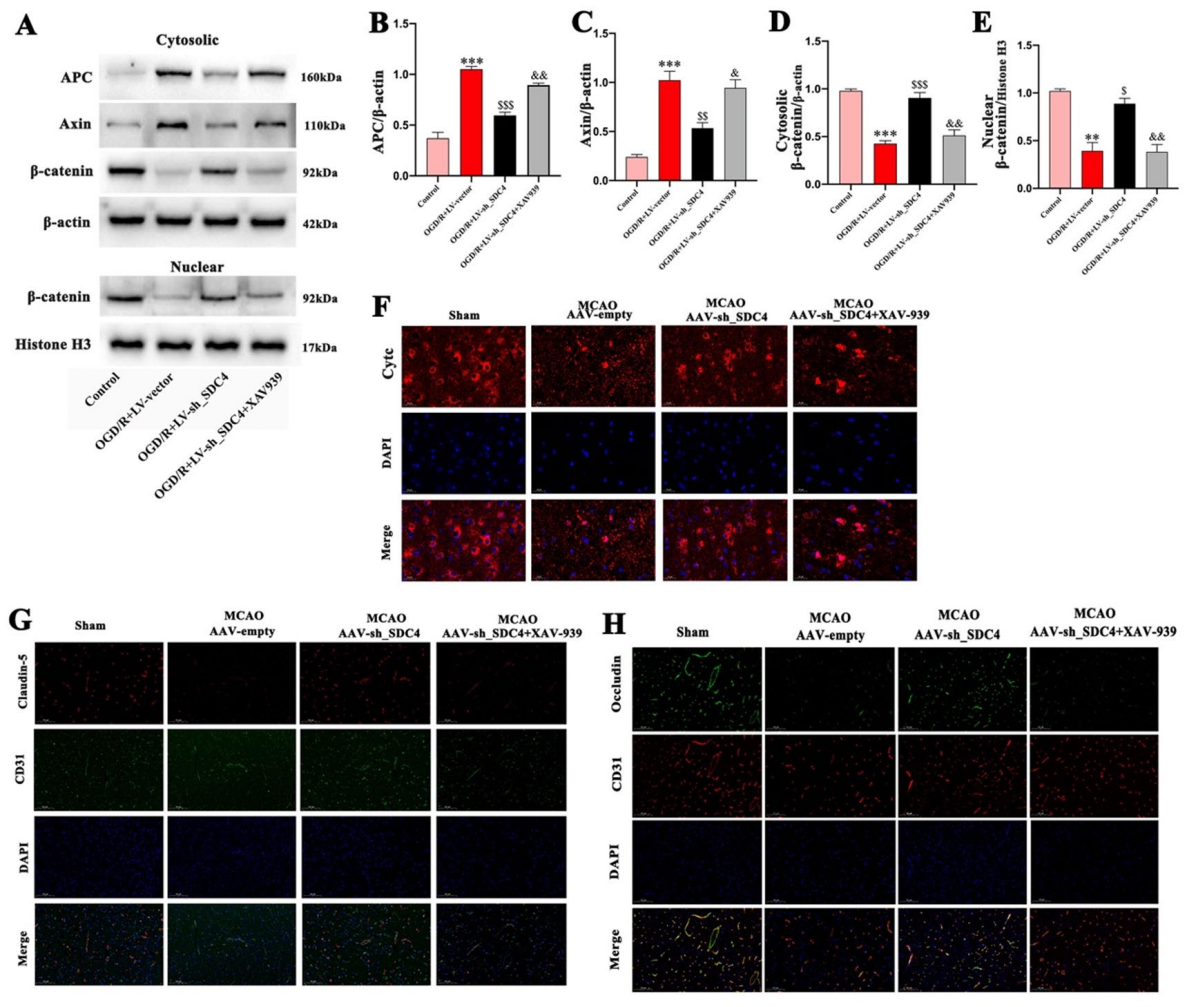
SDC4 contributes to BBB damage and mitochondrial integrity impairment following I/R injury by inhibiting the Wnt/β-catenin signaling pathway. (**A**–**E**) The protein expression and Western Blot analysis of APC, Axin and β-catenin in the microglia of different groups; *n* = 3. Data are expressed as mean ± SEM. and analyzed by one-way analysis of ANOVA with Bonferroni’s post hoc test. **p < 0.01, ***p < 0.001, as compared with the Control group; ^$^*p* < 0.05, ^$$^*p* < 0.01, ^$$$^*p* < 0.001, as compared with the OGD/R+LV-vector group; ^&^*p* < 0.05, ^&&^*p* < 0.01, as compared with the OGD/R+LV-sh_SDC4 group. F-H Representative Immunofluorescence staining of Cytc, Occludin and Claudin-5. Scale bar: 100 μm.

To further elucidate the mechanism by which SDC4 regulates the Wnt/β-catenin signaling pathway, we applied the specific pathway inhibitor XAV-939 to microglia after OGD. We subsequently detected the relevant proteins of the Wnt/β-catenin signaling pathway via Western Blot. The results showed that, compared to the control group, OGD/R significantly increased the expression of APC and Axin but decreased the levels of β-catenin in both the cytoplasm and nucleus. SDC4 interference downregulated the expression of APC and Axin in the cytoplasm and elevated the levels of β-catenin in both the cytoplasm and nucleus. However, XAV-939 treatment increased the levels of APC and Axin and reduced the levels of β-catenin in the cytoplasm and nucleus (Fig. 10A-E). The immunofluorescence staining results showed that XAV-939 promoted the release of Cyt c into the cytoplasm while suppressing Claudin-5 and occludin expression (Fig. 10F-H). In summary, these results indicate that SDC4 contributes to mitochondrial integrity impairment and is associated with BBB damage following I/R injury by inhibiting the Wnt/β-catenin signaling pathway.

### The simulations of molecular dynamics and calculations of binding free energy

**Fig. 11:**
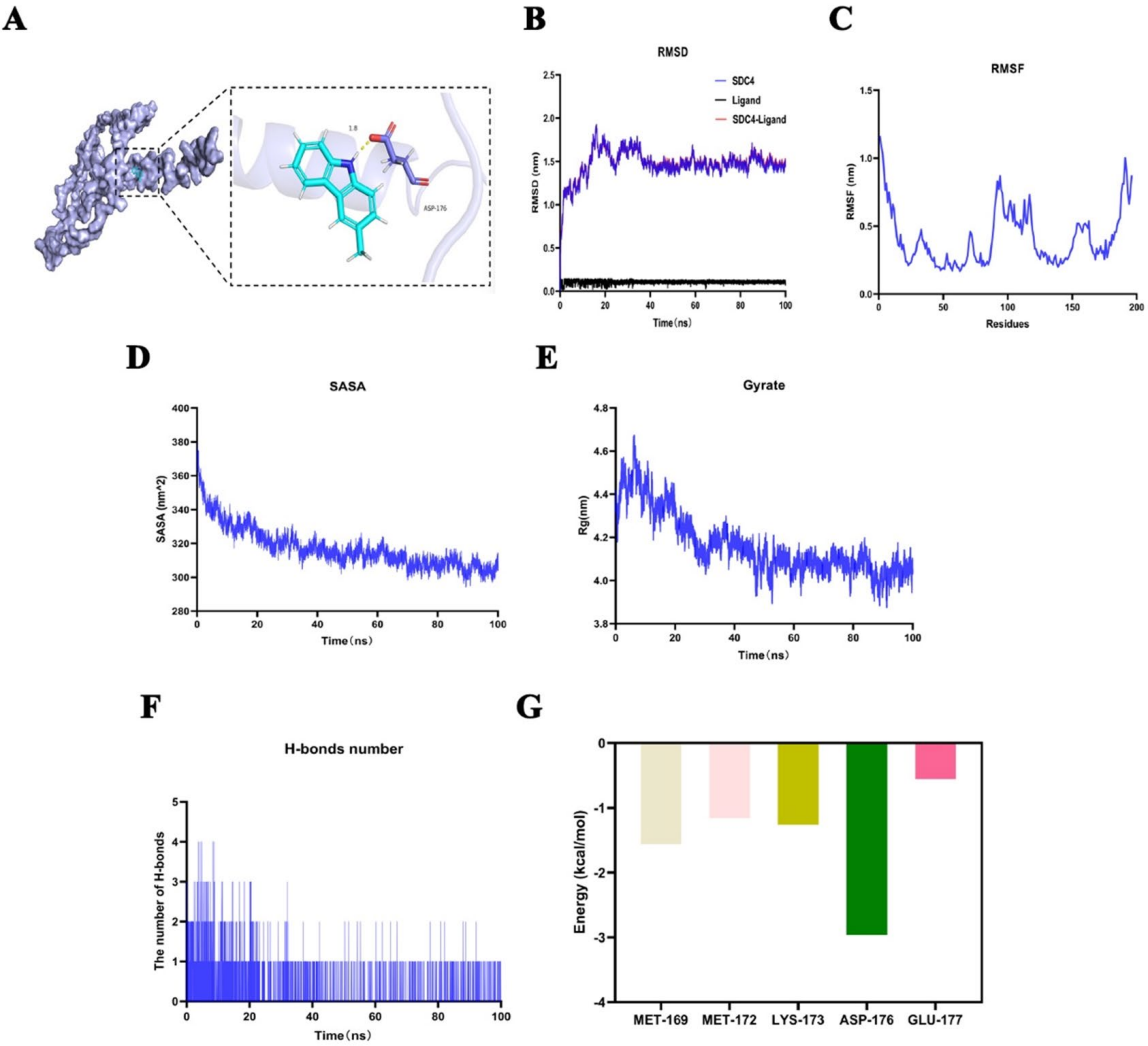
The analysis of MDS results. (**A**) The 3D binding mode of SDC4 with compounds. (**B**) RMSD. (**C**) RMSF. (**D**) SASA. (**E**) Rg. (**F**) HB profiles of the SDC4 with compounds systems. (**G**) Hot residue capacity contribution of the SDC4 with compounds systems.

Through the screening, we found that most of these compounds and proteins exhibit favorable binding energies (the smaller the binding energy value, the stronger the binding energy, generally less than − 5 indicates good binding energy) and good druggability. Subsequently, we selected the compound with the best binding energy in each group for 3D binding mode and interaction analysis with the protein. We investigated the binding mode and interactions between the target protein and the Top1 compound through molecular docking. As shown in the figure, the protein is depicted in blue, and the compound is represented by cyan sticks. Key amino acids are displayed as sticks, with red regions indicating oxygen atoms and dark blue regions indicating nitrogen atoms. Through docking, we found that the target protein and the compound both exhibit favorable binding energy. Additionally, the compound can interact with amino acids on the protein. Specifically, for the TOP1 compound, it can form one hydrogen bond interaction with ASP-176 on the protein. This interaction is crucial for the stability between the protein and the ligand, thereby significantly influencing their biological functions (Fig. 11A).

MD simulations were employed to investigate the dynamic interactions between the SDC4 protein and a Top1-targeted small-molecule ligand. To assess the complex’s stability and conformational dynamics, critical structural parameters including RMSD, RMSF, Rg, SASA, and hydrogen bond counts were analyzed. Simulation outputs revealed that the RMSD profile captured the overall stability and conformational shifts of the docked complex relative to the unliganded SDC4 protein (Fig. 11B). In the initial − 30 ns, both the free protein and ligand-bound complex displayed transient fluctuations-largely driven by the protein’s intrinsic flexibility-before reaching a steady state, confirming equilibrium under the applied MD conditions. Notably, the complex’s RMSD remained consistently below 0.5 nm throughout the stabilized phase.RMSF analysis quantified residue-level flexibility in the receptor protein (Fig. 11C). Regions with elevated RMSF peaks corresponded to more flexible segments-likely loop domains involved in ligand binding or proximal to functional active sites-suggesting these areas may enable dynamic ligand interactions and functional adaptability. Conversely, regions with low RMSF values denoted structurally rigid domains that preserve the complex’s overall integrity. SASA measurements evaluated the extent of protein-solvent interactions (Fig. 11D). Consistent SASA values across the simulation suggested that ligand binding did not substantially modify the protein’s solvent-exposed surfaces-a finding that may underpin the preservation of protein function and stability in the liganded state. Rg analysis gauged the complex’s compactness; a stable Rg profile implied the complex retained a consistent tertiary structure with no notable expansion or contraction, further confirming structural stability (Fig. 11E). Hydrogen bond analysis revealed the dynamic changes in hydrogen bond interactions between the target protein and the small molecule ligand throughout the simulation. Fluctuations in the number of hydrogen bonds during the simulation indicated transient yet sustained interactions between the target protein and the ligand. The stability of these interactions is crucial (Fig. 11F). We also analyzed the Hot residue energy contribution values (Fig. 11G), which showed that the amino acid residues contributing to the energy were MET-169, MET-172, LYS-173, ASP-176, and GLU-177. The MM/GBSA binding free energy analysis results are shown in the table, indicating that the complex exhibits favorable binding stability. The total binding free energy (ΔTotal) is -17.76 ± 1.82 kcal/mol, suggesting a certain degree of affinity between the two. From the perspective of energy components, the hydrophobic interaction (van der Waals force, ΔVDWAALS) contributes significantly, with a value of -16.22 ± 0.31 kcal/mol, indicating the potential existence of a stable hydrophobic embedding between the two. The electrostatic interaction (ΔEelec) is -10.85 ± 1.68 kcal/mol, also providing support for the stability of the complex. The polar solvation energy (ΔEGB) is positive (10.70 ± 0.60 kcal/mol), exerting a certain adverse effect on the binding. The non-polar solvation energy (ΔEsurf) is -1.39 ± 0.09 kcal/mol (Table 1). Overall, the binding between the two is primarily driven by hydrophobic interactions and electrostatic interactions, exhibiting a certain binding potential that may influence their conformational or functional states. The above results indicate that the Top1 compound can stably bind to the domain of SDC4 throughout the simulation, suggesting a strong binding stability between the two.

**Table 1:** Complex free energy distribution (kcal/mol).

Contribution components	
Δ_VDWAALS_	− 16.22 ± 0.31
ΔE_elec_	− 10.85 ± 1.68
ΔE_GB_	10.70 ± 0.60
ΔE_surf_	− 1.39 ± 0.09
ΔG_gas_	− 27.07 ± 1.71
ΔG_solvation_	9.31 ± 0.60
ΔTotal	− 17.76 ± 1.82

VDWAALS, van der waals energy; ELE, electrostatic interaction energy; EGB, polar solvation energy; ESURF: non-polar solvation energy; GGAS, total gas phase free energy; GSOLV: total solvation free energy; TOTAL: GSOLV + GGAS.

## Discussion

This study aims to explore how SDC4 contributes to mitochondrial dysfunction and its association with BBB damage, and to elucidate the underlying molecular mechanisms. The study demonstrates that SDC4 and the expression of SDC4 in microglia significantly increase after MCAO. SDC4 interference significantly alleviated MCAO-induced neurological dysfunction, which was associated with reduced inflammatory responses, improved mitochondrial function, and decreased infarct volume. Moreover, our findings demonstrate that SDC4 forms a direct physical interaction with Dvl, leading to the inhibition of the Wnt/β-catenin cascade and thus forging a distinct molecular connection between these two key molecules. Pharmacological inhibition of this cascade by XAV-939 partially abolished the protective effects of SDC4 knockdown on microglial mitochondrial function and BBB integrity suggesting that the Wnt/β-catenin cascade exerts a key regulatory effect on microglial activity and BBB structural integrity.

In this study, utilizing scRNA-seq data before and after MCAO, we identified 13 distinct cell types, including Endothelial cell, Oligodendrocyte, Mural cell, Vascular smooth-muscle cell, Microglial cell, Choroid plexus cell, Neuron, Ependymal cell, Fibroblast, Immune cell, Oligodendrocyte precursor cell, Astrocyte, and Proliferating cell. Among various cell types, microglia exhibit significant activation and proliferation after MCAO, which is crucial for the brain’s response to MCAO. Our findings show that microglia increase while endothelial cells relatively decrease during the acute phase after I/R injury, consistent with previous research results. Activated microglia can be classified into different subpopulations based on their response to injury or disease. Eight distinct microglial subpopulations were identified (three homeostatic clusters and five reactive clusters primarily emerging after ischemic stroke, referred to as Mic_M1L1, Mic_M1L2, Mic_np1, Mic_np2, and Mic_np3), including clusters associated with neurodegeneration and those with activated interferon pathways, highlighting the diverse roles microglia can assume in response to pathological conditions^42^. Our findings indicate that subpopulation analysis based on marker genes identified homeostatic, vasculature-associated, disease-associated, repair/anti-inflammatory, and aging-associated microglial subpopulations.

Broad and distinct alterations in gene expression over diverse temporal stages robustly mirror the transcriptional profiles linked to microglial transition. Shortly after injury (within minutes), microglia undergo activation; as the pathological process advances, these cells can further diversify into multiple phenotypic subtypes.As demonstrated by Wu et al., activation of microglia promotes M1 polarization-an event that amplifies neuroinflammatory processes and impairs neuronal activity^43^. M1 microglia further contribute to neuronal damage through excessive inflammatory responses. Our trajectory analysis revealed that homeostatic microglia appear at the beginning of the trajectory, repair/anti-inflammatory, disease-associated, and aging-associated microglia are primarily located in the center, and vascular-associated microglia emerge at the end of the trajectory, reflecting the gradual transformation of homeostatic microglia, repair/anti-inflammatory microglia, and aging-associated microglia into disease and vascular-associated microglia after I/R. We have further supplemented and validated previous research reports. In summary, the principal signaling pathways associated with homeostatic microglia, repair/anti-inflammatory, disease-associated, and aging-associated microglia influence the progression of MCAO.

Brain tissue and neural functions display a heightened susceptibility to injury resulting from MCAO^44^. After MCAO, the mice demonstrated an increase in cerebral infarct volume, a decline in motor coordination, and a reduction in muscle strength. In this study, we examined how SDC4 interference affects neural function in mice following MCAO by evaluating their performance on the rotarod test, mNSS scores, and grip strength measures. Our findings present initial evidence that the interference of SDC4 contributes to the recovery of neural function by improving motor coordination and enhancing muscle strength. Alongside the improvements in neural function, there was a notable decrease in the infarct area. Moreover, HE, Nissl, and TUNEL staining indicated that SDC4 interference significantly lessened neuronal damage post-MCAO. These outcomes suggest that the inhibition of SDC4 may mitigate cortical damage caused by MCAO and enhance neurological deficits stemming from I/R.

Cerebral ischemia typically induces damage to mitochondrial structure and function, which is closely associated with neuronal cell death^45,46^. Subsequent to I/R injury, perturbations in mitochondrial architecture within microglial cells become readily apparent. Overproduction of ROS disrupts mitochondrial membrane potential and undermines the structural integrity of mitochondrial membranes, ultimately resulting in neuronal apoptosis, degeneration, and cellular demise^47^.When cells undergo apoptosis, mitochondrial swelling, decreased density, alterations in cristae morphology, fusion of individual cristae, and widening of cristae junctions lead to the release of cyt c and AIF from mitochondria into the cytoplasm and mitochondrial dysfunction. Our findings indicate that SDC4 interference promotes the repair of mitochondrial structure reduces ROS levels induced by MCAO, and decreases the release of AIF and cyt c. These results suggest that SDC4 interference can maintain the integrity of microglial mitochondria after I/R, protecting mitochondrial structure and function.

The BBB’s disruption represents a critical pathological characteristic of ischemic stroke^48^. The inflammatory processes initiated by ischemic stroke contribute to this disruption, subsequently permitting external substances to penetrate the brain and inflict damage on brain tissues^49^. Vascular endothelial cells serve as the core structural component of the BBB, established through tight junctions interlinking the cells^50^. Research indicates that SDC4 is crucial for vascular endothelial functionality. Knockdown of HOXB9 suppressed the transcription of SDC4, which in turn dampened PKCα activation-ultimately mitigating BBB disruption, spinal cord injury (SCI), and blood-spinal cord barrier (BSCB) damage^19^. Our investigation reveals that interfering with SDC4 increases the expression of tight junction proteins in I/R mice. These findings illustrate that SDC4 interference bolsters the BBB’s integrity in I/R mice, aligning with the results reported by Wang et al.^19^.

As a highly conserved molecular cascade, the Wnt/β-catenin signal transduction cascade plays an essential role in modulating a broad spectrum of cellular processes, such as the suppression of apoptosis-a key biological mechanism that maintains cellular homeostasis and prevents aberrant cell death^51^. Upon binding of Wnt to frizzled (Frz), Frz acts on Dvl in the cytoplasm. Dvl proteins are capable of blocking the degradation cascade of β-catenin, which in turn facilitates the cytoplasmic accumulation and nuclear translocation of this protein. Conversely, in the absence of Wnt signaling, cytoplasmic β-catenin is recognized by a multiprotein complex comprising Axin, APC, and glycogen synthase kinase-3β (GSK-3β); following this recognition, GSK-3β mediates its phosphorylation, and the modified β-catenin is eventually targeted for degradation through the ubiquitin-proteasome system. Accumulating evidence from published studies indicates that activation of the Wnt/β-catenin pathway enhances BBB integrity by upregulating the expression of tight junction proteins, including occludin, claudin-5, and ZO-1, this improvement in BBB function, in turn, contributes to the recovery of neurological deficits in post-stroke mouse models^52^. Moreover, existing literature demonstrates that SDC4 engages in both biochemical and functional interactions with elements of the Wnt signaling cascade-including Dvl. Notably, Dvl exerts a critical influence on modulating the steady-state expression levels of SDC4. Increasing Dvl abundance amplifies Wnt5a-mediated SDC4 degradation, while inhibition of Dvl activity elevates SDC4 steady-state levels and diminishes its ubiquitination. Mutagenesis of the cytoplasmic lysine residue in SDC4 abrogates ubiquitination and results in elevated SDC4 cell surface expression during Xenopus gastrulation-suggesting that ubiquitination governs SDC4 dynamics in vivo. Consistent with these findings, our CO-IP assays reveal that SDC4 binds to Dvl following I/R injury in mice. Additionally, MD simulations and molecular docking analyses confirm a strong, stable binding interaction between SDC4 and Dvl.

At the cellular level, we extended our validation of SDC4 knockdown’s protective impact on microglia by modulating the Wnt/β-catenin signaling cascade. To dissect the molecular mechanisms linking SDC4 modulation to Wnt pathway activity, we initially employed XAV-939 (a selective inhibitor of the canonical Wnt/β-catenin axis). Our experimental findings revealed that SDC4 knockdown upregulates β-catenin expression levels; this observation implies that SDC4 inhibition confers cellular protection against OGD/R injury by triggering the canonical Wnt pathway. We next investigated the composition and dynamics of the β-catenin degradation complex. Notably, activation of the canonical Wnt cascade induces dissociation of this multiprotein complex, which comprises Axin, APC, and GSK-3β^53^. In the present study, we observed that SDC4 knockdown decreases the expression of the β-catenin degradation complex, and this effect is partially reversed by XAV-939 following OGD/R. Consistent with these functional observations, immunostaining micrographs (representative images shown) demonstrated that XAV-939 facilitates the translocation of Cyt c into the cytoplasm while downregulating claudin-5 and occludin expression. Our findings indicate that targeting the canonical Wnt/β-catenin signaling cascade holds promise as a therapeutic strategy for neuroprotection. SDC4 interference activated the Wnt/β-catenin signaling pathway and protected the integrity of the BBB and mitochondria. Additionally, the results of molecular docking indicate that the Top1 compound can stably bind to the domain of SDC4, demonstrating a strong binding stability between the two, which provides a theoretical basis for future drug development.

This study has several limitations. Due to reliance on single-cell sequencing data from public databases, the sample size is small, which restricts further experimental validation of other cell subtypes. Additionally, further construction of a conditional knockout model of SDC4 could enhance the accuracy of the conclusions. Furthermore, while our in vitro data support the specific role of SDC4 in microglia, establishing microglia-specific conditional knockout models (e.g., CX3CR1-CreERT2 mice) would provide definitive in vivo evidence. We plan to address this issue in future studies to further validate our conclusions.

## Conclusion

The present study reveals that SDC4 knockdown enhances neurological recovery in mouse models of MCAO through suppressing two key pathological processes: mitochondrial dysfunction in microglia and BBB impairment. Specifically, we observed that the interaction between SDC4 and Dvl leads to the sequestration of Dvl, which subsequently inhibits the Wnt/β-catenin signaling pathway following MCAO. This inhibition contributes to motor function deficits, mitochondrial dysfunction, and associated BBB damage. The present study reveals that SDC4 interference serves as a potential therapeutic strategy for the treatment of ischemic stroke.

## Data Availability

The datasets used and/or analysed during the current study are available from the corresponding author on reasonable request.
